# Supplementary material for: Predicting asthma attacks in primary care: protocol for developing a machine learning-based prediction model
Source: BMJ Open. 2019 Jul 9;9(7):e028375. doi: 10.1136/bmjopen-2018-028375 (PMC6624024; doi:10.1136/bmjopen-2018-028375)
Supplement: Supplementary file 3 [file bmjopen-2018-028375supp003.docx]

In this publication, I explain how I am going to build a computer program to predict how likely someone is to have an asthma attack in the next week. This will be done using data collected by the GP.

Asthma can be very unpredictable, and it can be very hard to tell when an attack might happen. Having lots of symptoms does not always mean the person has lots of asthma attacks – some people are more likely to have attacks than others. We know that irregular use of a preventer inhaler (or other treatment), smoking, obesity, history of chest infections, mean you are at a higher risk of attacks. Even though we know about so many risk factors we cannot tell who is actually going to have an attack.

We can use data collected routinely during GP consultations with our program, which uses **machine learning algorithms**, to estimate asthma attacks. Machine learning algorithms are good at identifying different combinations of risk factors (called **interactions**) which are important, instead of looking at them individually. Doing this can estimate very accurately the risk of an attack. These methods need a lot of data and computer power. This is why we are using data that is collected regularly at all GP visits, and very powerful computers.

The number of people having an asthma attack is a very small percentage of all those with asthma. If 5% of people with asthma have an attack during a study, then we could predict whether an attack would occur correctly 95% of the time by saying simply that NO ONE would have an attack. But this statistic is not very helpful. One way we can improve prediction when the event we are trying to predict is rare, is by changing the data we use to build the model. This is known as **data enrichment.** One method of enrichment, for example, is to take out some of the people who had no asthma attacks so that the proportions of people that did and did not have attacks are more balanced. We are going to try a range of enrichment methods and see which is most appropriate in this setting.

Being able to predict attacks accurately means both that preventative care can be provided to stop the attack happening, and that unnecessary additional treatments can be avoided if an attack is unlikely. This could reduce the amount of oral steroids that someone with a high recurring risk of attacks might need over a lifetime, which could reduce the likelihood of steroid related side effects.
