# Supplementary material for: Predicting asthma attacks in primary care: protocol for developing a machine learning-based prediction model
Source: BMJ Open. 2019 Jul 9;9(7):e028375. doi: 10.1136/bmjopen-2018-028375 (PMC6624024; doi:10.1136/bmjopen-2018-028375)
Supplement: Supplementary file 1 [file bmjopen-2018-028375supp001.pdf]

Appendix A – Data harmonisation plan

Variable	Derivation dataset (LHS) format	External (unseen; SIVE II) dataset format	Harmonised format
Sex	Character – "M", "F" and "I" (less than 0.001% of records)	Character – "M", "F"	Character – "M", "F" and "I"
Birthday	Age (integer) at data extraction date (31st March 2018) or deduction date (indicated)	YYYY-MM-DD date format, all days set to 01 (true day redacted)	Age on March 31st, 2015 (approximate)
Scottish Index of Multiple Deprivation	Quintiles, 2012 and 2009 values	Deciles, 2012 values	Quintiles, 2012 values
Scottish Government Urban Rural Classification Scale	6-fold scale, from (1) Large Urban Areas to (6) Remote Rural Areas	8-fold scale, from (1) Large Urban Areas to (8) Very Remote Rural Areas	6-fold scale, from (1) Large Urban Areas to (6) Remote Rural Areas, 8-fold scale recoded as follows:<br>1 > 1<br>2 > 2<br>3 > 3<br>4, 5 > 4<br>6 > 5<br>7,8 > 6
Cause of death	ICD10 coded primary field, and 10 secondary cause fields	ICD10 coded primary field, and 10 secondary cause fields	*Aligned*
A&E cause of presentation	Presenting complaint free text field and 3 ICD10 coded disease fields	Presenting complaint free text field and 3 ICD10 coded disease fields	*Aligned*
Primary care records	Read Codes (version 2)	Read Codes (version 2)	*Aligned*
Primary care prescriptions	Standardised [a] text drug name and dose fields	Standardised [a] text drug name and dose fields	*Aligned*
Hospital inpatient admission records	N/A	ICD10 coded primary field, and 5 secondary cause fields	*Omitted as alignment not possible*
Event Date	Standardised date format	Standardised date format	*Aligned*

[a] Auto-fill assisted free text field
