# Supplementary material for: Predicting asthma attacks in primary care: protocol for developing a machine learning-based prediction model
Source: BMJ Open. 2019 Jul 9;9(7):e028375. doi: 10.1136/bmjopen-2018-028375 (PMC6624024; doi:10.1136/bmjopen-2018-028375)
Supplement: Supplementary file 2 [file bmjopen-2018-028375supp002.pdf]

Appendix B – Machine Learning classifier hyperparameters

Naïve Bayes Classifier
Implemented using the r function *naivebayes*, from the package of the same name [72].
No hyperparameters.

Support Vector Machine
Implemented using the r function *svm*, from the package *e1071* [73] *which builds upon the LIBSVM package* [74], using a radial basis kernel function.

- *GAMMA* = Radial basis kernel function gamma parameter, corresponding to the kernel bandwidth (default 1/k): $2^{(-5:10)}$
- *COST* = Cost of constraints violation, i.e. samples penalised when crossing the boundary (default 1): $2^{(-5:10)}$

Ensemble: Bagging
Bagging methods learn from multiple models which are staged in parallel.
Random Forests
Implemented using the r function *randomForest*, from the package of the same name [75].

- *NTREE* = Number of trees to grow (default 500): 500, 750, 1000
- *MTRY* = Number of variables randomly sampled as candidates at each split (default square root of the number of predictors; k): floor($0.5 * \sqrt{k}$), floor($\sqrt{k}$), floor(2*$\sqrt{k}$) – in which floor represents the rounded-down integer value.

Ensemble: Boosting
Learning from multiple models which are staged *sequentially*, usually tree-based, constructed from different subsamples of the training dataset.
Extreme Gradient Boosting
Implemented using the r package *xgboost* [76], with 10-fold cross validation, repeated 3 times.

- NROUNDS = maximum number of iterations (default 100): 50,100
- MAXDEPTH = Maximum depth of each tree (default = 6): (1:5)^2
- ETA = step size of each boosting step (default = 0.3): 0.25, 0.5, 1

Ensemble: Stacking
Combining models from different classifiers, with an over-arching supervisor model which determines the best way to use all sources of information for prediction. The base set of weak learners will comprise all aforementioned model and hyperparameter combinations, and the meta-learner (random forest with 500 trees and mtry = floor($0.5 * \sqrt{k}$)) will use all weak learners with a validation set performance in the top 50%.
