# Supplementary material for: Predicting asthma attacks in primary care: protocol for developing a machine learning-based prediction model
Source: BMJ Open. 2019 Jul 9;9(7):e028375. doi: 10.1136/bmjopen-2018-028375 (PMC6624024; doi:10.1136/bmjopen-2018-028375)
Supplement: Author's manuscript [file bmjopen-2018-028375.draft_revisions.pdf]

BMJ Open

BMJ Open is committed to open peer review. As part of this commitment we make the peer review history of every article we publish publicly available.

When an article is published we post the peer reviewers' comments and the authors' responses online. We also post the versions of the paper that were used during peer review. These are the versions that the peer review comments apply to.

The versions of the paper that follow are the versions that were submitted during the peer review process. They are not the versions of record or the final published versions. They should not be cited or distributed as the published version of this manuscript.

BMJ Open is an open access journal and the full, final, typeset and author-corrected version of record of the manuscript is available on our site with no access controls, subscription charges or pay-per-view fees (http://bmjopen.bmj.com).

If you have any questions on BMJ Open's open peer review process please email info.bmjopen@bmj.com

BMJ Open

Predicting asthma attacks in primary care: protocol for developing a machine learning-based prediction model

Journal:	*BMJ Open*
Manuscript ID	bmjopen-2018-028375
Article Type:	Protocol
Date Submitted by the Author:	07-Dec-2018
Complete List of Authors:	tibble, holly; University of Edinburgh School of Molecular Genetic and Population Health Sciences; Asthma UK Centre for Applied Research<br>Tsanas, Athanasios; University of Edinburgh; Asthma UK Centre for Applied Research<br>Horne, Elsie; University of Edinburgh; Asthma UK Centre for Applied Research<br>Horne, Robert; University College London; Asthma UK Centre for Applied Research<br>Mizani, Mehrdad; University of Edinbugh; Asthma UK Centre for Applied Research<br>Simpson, Colin; Victoria University of Wellington, School of Health; Asthma UK Centre for Applied Research<br>Sheikh, Aziz; University of Edinburgh, Division of Community Health Sciences; Asthma UK Centre for Applied Research
Keywords:	Asthma < THORACIC MEDICINE, PRIMARY CARE, Asthma Attacks, Machine Learning, Prediction

SCHOLARONE™
Manuscripts

Predicting asthma attacks in primary care: protocol for developing a machine learning-based prediction model

Word Count 2908/4000

Authors:
Holly Tibble [1,2] (corresponding author), Athanasios Tsanas [1,2], Elsie Horne [1,2], Rob Horne [2,3], Mehrdad A. Mizani [1,2], Colin R. Simpson [4,2], Aziz Sheikh [1,2]

1. Usher Institute of Population Health Sciences and Informatics, Edinburgh Medical School, College of Medicine and Veterinary Medicine, University of Edinburgh – Teviot Place, Edinburgh, United Kingdom
2. Asthma UK Centre for Applied Research, Usher Institute of Population Health Sciences and Informatics, Centre for Medical Informatics, The University of Edinburgh, Edinburgh, UK
3. Centre for Behavioural Medicine, Department for Practice and Policy, UCL School of Pharmacy, University College London – Mezzanine Floor, London, United Kingdom
4. School of Health, Faculty of Health, Victoria University of Wellington, Wellington, New Zealand

Author's Email Addresses:
Holly.tibble@ed.ac.uk
Athanasios.Tsanas@ed.ac.uk
Elsie.Horne@ed.ac.uk
r.horne@ucl.ac.uk
Mehrdad.Mizani@ed.ac.uk
c.simpson@ed.ac.uk
Aziz.Sheikh@ed.ac.uk

Author Contributions:
HT and AT conceived and planned the analysis. HT wrote the first draft, with contributions from all authors. All authors approved the final version and jointly take responsibility for the decision to submit this manuscript to be considered for publication.

Conflicts of Interest:
None to report

Keywords:
Asthma, Asthma Attacks, Primary Care, Machine Learning, Prediction

Acknowledgements:
The authors would like to thank Eleftheria Vasileiou, Amy Tilbrook, Mome Mukherjee and Jill Tibble for their contributions to the analysis plan, data management, and proof-reading of this manuscript, and the AUKCAR Patient and Public Involvement group for their contribution to the analysis plan.

For peer review only

ABSTRACT

Introduction

Asthma is a long-term condition with rapid onset worsening of symptoms which can be unpredictable and fatal. Prediction models require high sensitivity to minimise mortality risk, and high specificity to avoid unnecessary prescribing of preventative medications that come with a risk of adverse events. We aim to create a risk score to predict asthma attacks in primary care using a statistical learning approach trained on routinely collected Electronic Health Record (EHR) data. We will investigate the potential added value across various metrics (including sensitivity and specificity) by extending the statistical learning model, incorporating information extracted from linked secondary care records in addition to the primary care EHR data.

Methods and Analysis

We will employ various machine learning classifiers (such as naïve Bayes, support vector machines, and random forests) to create an asthma attack risk prediction model, using the Learning Health System study patient registry comprising 500,000 individuals from across 75 Scottish general practices, with linked longitudinal primary care prescribing records, primary care Read codes, accident and emergency records, hospital admissions and deaths. Models will be compared on a partition of the dataset reserved for validation, and the final model will be tested in both an unseen partition of the derivation dataset and in an external dataset (from the Seasonal Influenza Vaccination Effectiveness II study).

Ethics and Dissemination

Permissions for the LHS project were obtained from the South East Scotland Research Ethics Committee 02 [16/SS/0130] and the Public Benefit and Privacy Panel for Health and Social Care [1516-0489]. Permissions for the SIVE II project were obtained from the Privacy Advisory Committee (National Services NHS Scotland) [68/14] and the National Research Ethics Committee West Midlands - Edgbaston [15/WM/0035]. Code scripts used for all components of the data cleaning, compiling, and analysis will be made available in the open source GitHub website.

For peer review only

ARTICLE SUMMARY

Strengths and Limitations of this study

- Large and representative sample size of over 500,000 individuals: people from 75 general practices in Scotland recruited
- Novel application of established machine learning methodologies
- Prediction model tested in unseen external dataset collected from a different research group
- Developed in NHS Scotland only; generalisability in other UK National Health Services and international health systems is untested

FUNDING STATEMENT

HT is supported by College of Medicine and Veterinary Medicine PhD (eHERC/Farr Institute) Studentships from The University of Edinburgh. EH is supported by a Medical Research Council PhD Studentship (eHERC/Farr). MAM's Newton International Fellowship is awarded by the Academy of Medical Sciences and Newton Fund. This work is carried out with the support of the Asthma UK Centre for Applied Research [AUK-AC-2012-01] and Health Data Research UK, an initiative funded by UK Research and Innovation Councils, National Institute for Health Research (England) and the UK devolved administrations, and leading medical research charities. The LHS dataset was created with funding from the National Environment Research Council [NE/P011012/1]. The SIVE II dataset was created with funding from the National Institute for Health Research (NIHR) Health Technology Assessment programme [13/34/14] - the views and opinions expressed therein are those of the authors and do not necessarily reflect those of the Health Technology Assessment programme, NIHR, NHS or the Department of Health.

For peer review only

INTRODUCTION

Asthma is a long-term lung disease characterised by inflammation of the airways, which may manifest as episodic wheezing, chest tightness, coughing and shortness of breath. An asthma attack is the sudden worsening of symptoms, which may prove fatal [1]. In 2017, asthma was estimated to affect 235 million people worldwide [2]. In 2015 alone, 1,434 people died from asthma attacks in the United Kingdom (UK) – a rate of 2.21 deaths per 100,000 person-years [3].

Asthma therapy typically follows a fairly linear path – beginning with a short-acting bronchodilator in those without persistent asthma symptoms, and adding preventative treatments and long-acting bronchodilators in those with more persistent asthma symptoms [4,5]. Those with persistent troublesome symptoms and/or considered to be at very high risk may be prescribed biologicals and/or oral steroids [6]. Oral steroids are often considered a last resort, due to their undesirable safety profile including increased risk of diabetes [7–9], osteoporosis [10–12], and psychotic and affective disorders [12–15].

It follows that the determination of those at high risk for asthma attacks is crucial in order to prevent attacks and minimise the risk of side-effects. Furthermore, the 2014 National Review of Asthma Deaths found that 45% of asthma deaths in the study year died without requesting medical help, or before that help could be provided [16]. Increased awareness of the risk could prevent those with asthma from delay in seeking medical care and preventing fatality.

While it might seem intuitive that those with the most severe asthma, i.e. those with continuous symptoms that are not controlled by medication, exhibit greater risk of severe morbidity and mortality, research suggests that daily symptoms may be a suboptimal clinical marker of disease severity [17]. Indeed, some people with asthma are more prone to attacks than others, with past attack history being commonly found to be one of the strongest risk factors for future attacks [18–21]. Other commonly identified risk factors for asthma attacks include poor asthma control [22–25] (often a result of poor adherence to preventative therapy [26–29]), smoking [22,25,30–32], history of hospital admission [19,22], history of oral steroid use [22], obesity [25,32–36], socio-economic factors such as access to medicines [37,38] socioeconomic status [39,40], and viral respiratory infections [41–43].

Despite the identification of many risk factors, identifying high risk patients has proven a challenging task. Logistic regression, the most commonly used statistical method for event prediction, is known to predict outcomes poorly when class sizes (event and no event) are imbalanced [44]. As such, most prediction models report high *specificity* (correctly predicting low attack risk to those who did not have attacks) but low *sensitivity* (correctly predicting high risk in those who did go on to have attacks) [22,39,45–50], which results in less reliable risk prediction for the most at risk patients.

In a recent study by Finkelstein and Jeong [51], sensitivity (and specificity) in excess of 75% was achieved for all classifiers (Adaptive Bayesian network, Naïve Bayes classifier, and Support Vector Machine) predicting asthma attacks a week in advance using a sample of just over 7000 records of home tele-monitoring data. They found substantial improvements in model sensitivity using *training enrichment* methods; pre-processing the training data to improve the performance in the testing data – in this case, modifying the prevalence of the outcome in the training data by stratifying samples to balance the classes.

For peer review only

RESEARCH AIM

We aim to create a risk score for primary care clinicians to predict asthma attacks in the following 1, 4, 26, and 52 weeks, employing machine learning methodologies such as random forests, naïve Bayes classifiers, and support vector machines, as well as ensemble algorithms. Secondly, we aim to explore the potential added value of possible future routine data linkages, such as secondary care records, to investigate improved ability to predict asthma attacks.

METHODS

Data Sources and Permissions

The derivation dataset used for training, validating, and testing the model will be the Asthma Learning Healthcare System (LHS) dataset, created in order to develop and validate a prototype learning health system for asthma patients in Scotland [52]. The LHS study aimed to increase understanding of variation in asthma outcomes and create benchmarks for clinical practice in order to reduce sub-optimal care, by repurposing patient data to create a continuous loop of knowledge-generation, evidence based clinical practice change, and change assessment. The study dataset contains patient demographics from the patient registry, primary care prescribing records, primary care encounters, Accident and Emergency (A&E) records, hospital inpatient admissions and deaths, linkable by an anonymised identifier. Datasets were extracted between November 2017 and August 2018 for the period January 2000 to December 2017, as shown in Table 1, along the number of records and unique individuals before data cleaning.

In order to externally verify the prediction model, we will evaluate its performance using an external cohort study dataset, the second Seasonal Influenza Vaccination Effectiveness (SIVE II) cohort study [53,54], which used a large national primary care (1.25 million individuals from 230 Scottish general practices) and laboratory-linked dataset to evaluate live attenuated and trivalent inactivated influenza vaccination effectiveness. The dataset contains records from the same sources (primary and secondary care) and modalities (diagnosis and date) as the LHS dataset (extraction and specification dates are shown in Table 2), and as such can be harmonised such that variables and value sets are aligned. In Appendix A, we detail the data harmonisation plan, that is, we list the key variables to be used in the following analyses, their format in each dataset (for example, whether age is pre-coded into 5-year bands) and the common denominator format that will be used in the analyses to ensure the highest degree of concordance during the validation stage.

Permissions for the LHS project were obtained from the South East Scotland Research Ethics Committee 02 [16/SS/0130] and the Public Benefit and Privacy Panel for Health and Social Care [1516-0489]. Permissions for the SIVE II project were obtained from the Privacy Advisory Committee (National Services NHS Scotland) [68/14] and the National Research Ethics Committee West Midlands - Edgbaston [15/WM/0035].

Patient and Public Involvement

This analysis plan was constructed with the assistance of the Asthma UK Centre for Applied Research (AUKCAR) Patient and Public Involvement (PPI) group. The particular focus in this research to reduce preventative steroid prescribing, where possible, was a result of discussions within this group about the burden of treatment side-effects. For their support and advice, we

For peer review only

are very grateful. A lay summary of the results of this study will be disseminated after publication.

Inclusion Criteria

We will identify our study population as all individuals with asthma identified by clinical diagnoses (Read codes) and relevant prescribing records in primary care. Patients with missing sex or age information will be removed; this and any other patient exclusions from further analysis will be explicitly detailed.

All records from the derivation dataset (LHS) will be left-censored at January 2010, in order to align with the primary care prescribing data, and right-censored at March 2017, in order to align with the mortality, primary care Read code, and inpatient hospital admission records, are presented in Table 1. Similarly, records from the external dataset (SIVE II) will be left-censored at January 2003, in order to align with the primary care prescribing data, and right-censored at August 2016 to align with the A&E records, as shown in Table 2. There is a high probability that some individuals having been recruited into both studies, and so such individuals will be flagged in the external testing dataset and removed from the study pool.

Table 1: Meta-data for Clinical Data sources in Derivation Dataset (LHS)

Data Source	Number of Records	Number of Individuals	Extraction Date	Data Specification Date Range
Primary Care Prescribing [a]	6,886,922	54,565	March 2018	January 2010 – December 2017
Primary Care Encounters [a]	11,766,100	49,307	March 2018	January 2000 – November 2017
Accident & Emergency	1,831,789	500,321	November 2017	June 2007 – September 2017
Hospital Inpatient Admissions	1,668,957	342,838	August 2018	January 2000 – March 2017
Mortality	*NA*	91,758	May 2018	January 2000 – March 2017

a. Records available for subset of study population with asthma diagnosis only

Table 2: Meta-data for Clinical Data sources in External Dataset (SIVE II)

Data Source	Number of Records	Number of Individuals	Extraction Date	Data Specification Date Range
Primary Care Prescribing	29,360,448	1,073,377	May 2017	January 2003 – March 2017
Primary Care Encounters	31,878,423	1,887,957	May 2017	January 2000 [a] – March 2017
Accident & Emergency	4,116,561	1,247,314	April 2017	June 2007 - August 2016
Hospital Inpatient Admissions	3,549,174	794,937	April 2017	January 2000 - March 2017

For peer review only

Mortality	*NA*	215,466	April 2017	January 2000 - March 2017

[a] *Diagnosis codes entered in this period, but post-dated from 1940 onwards retained.*

Outcome Ascertainment

We will identify asthma attacks, defined by the American Thoracic Society/European Respiratory Society [55] as either a prescription of oral corticosteroids, an asthma-related A&E visit, or an asthma-related hospital admission. Additionally, deaths occurring with asthma as the primary cause will be labelled as asthma attacks. Instances of multiple attack indicators occurring within a 14-day period were coded as a single attack.

Patient characteristics, confounders, and missing data handling

Patient characteristics will be presented at baseline and included as confounders in analyses. For all characteristics derived from Read codes, full code lists will be provided as supplementary materials.

Demographics: Age, sex, rurality, and social deprivation will be extracted from the primary care registry. Social deprivation is measured using quintiles of the Scottish Index of Multiple Deprivation (SIMD), a geographic measure derived using data on income, employment, education, health, access to services, crime and housing [56]. Rurality is defined using the Scottish Government Urban Rural Classification Scale (6-fold scale) [57]. While missing age and/or sex are exclusion criteria for the study sample, missingness for rurality and social deprivation within the registry will be coded as 'missing'.

Practice Location: Practice location will be included in order to account for clustering of patients by region. Location will be coded using the Nomenclature of Territorial Units for Statistics [58] (NUTS 3) codes, linked from the registered practice data zone (2001) available in the patient registry.

Asthma Severity: Patient asthma severity will be categorised using the British Thoracic Society's 2016 5-step treatment classification [59]. Severity will be considered time-dependent and will be determined using prescribing records at any change in regimen.

Smoking Status: Smoking status will be derived from primary care data, and presented as a 3-level variable, namely: current, former, and non-smoker, using the most recent smoking Read code at any day. Those with unknown smoking status will be coded as non-smokers [60,61]. Smoking status will be considered time-dependent and determined using the most recent Read code records at the start of each study year.

Blood Eosinophil Count: Blood eosinophil count will be derived from primary care Read codes, and will be dichotomised at >=400 cells per µL. Those with unknown Blood eosinophil count will be coded as negative for raised eosinophil count. Blood eosinophil count will be considered time-dependent and determined using the most recent Read code records at the start of each study year.

Obesity: Obesity will be derived from Body Mass Index (BMI) recordings in primary care data, and will be presented as a binary variable (BMI≥30). Those with unknown BMI will be

For peer review only

coded as non-obese. Obesity will be considered time-dependent and determined using the most recent Read code records at the start of each study year.

Comorbidity: Comorbidity will be defined by 17 dichotomous (unweighted) variables representing the diagnostic categories of the adapted Charlson Comorbidity Index [62,63]. Additionally, active diagnoses of rhinitis, eczema, Gastroeosophageal Reflux Disease (GERD), nasal polyps, and anaphylaxis will be recorded; all identified by Blakey et al. as contributing characteristics to increased asthma attack risk [64]. Comorbidities will be considered time-dependent and determined using Read code records prior to the start of each study year.

Previous Healthcare Usage: The number of repeat prescriptions of preventer medication, and the number of primary care asthma encounters (days on which at least one asthma related code was recorded) in the previous year will be derived from primary care prescribing and Read code records, respectively. Both will be considered time-dependent and determined using records from the previous calendar year.

Asthma Control: The mean Short-Acting Beta-2 Agonist (SABA) dose per day will be estimated retroactively by examining the dates between prescriptions. The most recent peak expiratory flow measurement at any time will be recorded (categorical, based on percentage of previous maximum) or coded as missing if that measurement was more than seven days ago. Adherence to preventer therapy will be approximated using the medication possession ratio, calculated from primary care prescribing records.

History of Asthma Attacks: Asthma attacks will be identified using both primary care prescribing records and secondary care records for outcome ascertainment. Prior asthma attacks will also be used as a predictor, however, and for this purpose will be identified from primary care prescribing records and primary care Read codes only. This is because primary care practitioners will not be able to make use of secondary care records when utilising this risk score with patients. Both the prior number of attacks, and the time since the last attack, will be included as predictors and will be considered time-dependent and accurate at the daily level.

Analysis Plan

A multivariate repeated-event survival analysis will be used to assess the contributing risk factors of time-to-asthma-attack, consisting of static demographic variables and time-varying data such as season and historical asthma records.

The derivation dataset (LHS) will be divided into three partitions: 60% for training, 20% for model comparison (validation), and 20% to assess performance (testing). In our training subset, the first partition, we will train machine learning models (classifiers) with varying hyperparameters, predicting asthma attack occurrence in the following 1, 4, 26, and 52 weeks. We will run 100 iterations for statistical confidence, each time randomly permuting samples prior to determining the three subsets. The classifiers employed will include random forests, naïve Bayes classifiers, and support vector machines, as well as ensemble learning using combinations of these models.

A selection of *training enrichment* methods will be trialled, in order to assess how to best overcome poor performance as a result of low outcome prevalence. Typically, modelling rare events results in reduced sensitivity (the proportion of those who had attacks that were

For peer review only

detected), so those predicted to be low-risk will have a high rate of asthma attacks. As such, this start of this process (the first 20 iterations of training each model) will be repeated five times, using:

1. the raw data,
2. raw data + duplicates of the positive outcome records (a method known as *over-sampling* [65]),
3. raw data – a selection of the negative outcome records (*under-sampling* [65]),
4. raw data + slightly modified duplicates of the positive outcome records – a selection of the negative outcome records (*Synthetic minority over-sampling; SMOTE* [65,66]),
5. raw data, using the outcome classification threshold to maximise the MCC metric – identified using golden-section search optimisation [67].

By assessing the average performance, by classification method class, in each set of iterations, we will determine which enrichment method is the most appropriate overall for the data, and continue accordingly.

In the validation partition, with all 100 iterations for the selected enrichment methods, we will compare the performance of each trained model, using our primary metric – the Matthew's Correlation Coefficient (MCC) [68]. From here, the performance of each model within an iteration will be ranked. Across iterations, the highest performing model will be selected as follows:

1. Models with a median MCC (across iterations) lower than the 90th percentile for all models and iterations will be removed;
2. The model with the highest mean MCC (across iterations) is selected; in the event of a tie, the model with the highest worst-performing iteration will be selected.

Model testing will be conducted on the selected model (Figure 1) in the derivation testing partitions. Model calibration will be assessed by comparing observed rate of incidence by predicted risk, for the full population and by exhaustive population subgroups. Performance in the testing datasets will be assessed using the MCC, and the additional metrics of sensitivity, specificity, positive predictive value and negative predictive value, and the F_1 measure [69], along with information criteria such as the Bayesian Information Criterion (BIC) to obtain a trade-off between model complexity and accuracy. Confusion matrices (also known as contingency tables) will be made available as supplementary materials.

The derivation dataset will be re-used in its entirety to retrain the model based on the final classifier and hyperparameter selection. Model testing will then be conducted in the external dataset, which consists of data unseen in the model derivation, using this trained model. Distributions of predictors between the derivation and external datasets will be assessed (indirectly) to contextualise the generalisability findings. The aforementioned metrics will be reported.

Finally, we will re-train the derivation dataset using the hyperparameter specifications from the best performing model, and incorporating data extracted from secondary care records (such as A&E presentations for asthma attack not captured in primary care records), in order to evaluate the added value of secondary care data linkage for this predictor, determined by the same metrics used for model evaluation.

For peer review only

All analyses will be conducted in R (though the RStudio interface), and details on the functions, the hyperparameter within each classifier, and the ranges assessed herein, are provided in Appendix B.

[[INSERT FIGURE 1 HERE]]

Figure 1: Process of selecting the highest performing model from the validation data, and the average performance of this model across iterations in the testing dataset. In the foreground we have the first iteration. We will use 100 iterations for statistical confidence, randomly permuting the data into training, validation, and testing subsets in each iteration

Ethics and Dissemination

All authors with data access have completed the Safe Users of Research data Environment (SURE) training, provided by the Administrative Data Research Network (ADRN). All analysis will be conducted in concordance with the National Services Scotland Electronic Data Research and Innovation Service (eDRIS) user agreement. This study protocol will be registered with the European Union electronic Register of Post-Authorisation Studies (EU PAS Register) as a non-interventional post-authorisation study (PAS) before any data analysis is initiated.

The subsequent research paper will be submitted for publication in a peer-reviewed journal and will be written in accordance with TRIPOD: *transparent reporting of a multivariable prediction model for individual prognosis or diagnosis* [70] and RECORD: *reporting of studies conducted using observational routinely-collected health data* [71] guidelines. Code scripts used for all components of the data cleaning, compiling, and analysis will be made available in the open source GitHub website.

Data statement

The derivation and external datasets used in this study are accessible via the eDRIS secure platform under the project numbers 1516-0160 and 1516–0489, respectively.

Conclusions

This project will further advance asthma attack risk prediction modelling and will inform on the future direction of routine data linkage in Scotland, which is likely to have additional benefits for other health systems in the United Kingdom and internationally.

For peer review only

References

1. Bush, A. & Griffiths, C. Improving treatment of asthma attacks in children. *BMJ (Online)* (2017). doi:10.1136/bmj.j5763
2. World Health Organisation. *Asthma Fact Sheet (2017). World Health Organisation Fact Sheets* (World Health Organization, 2017).
3. Asthma UK. *UK asthma death rates among worst in Europe*. (2017).
4. British Thoracic Society & Scottish Intercollegiate Guidelines Network. British guideline on the management of asthma. *SIGN Guidel.* (2014). doi:10.1136/thx.2008.097741
5. Currie, G. P., Douglas, J. G. & Heaney, L. G. Difficult to treat asthma in adults. *BMJ* **338,** b494 (2009).
6. British Thoracic Society, Research Unit of the Royal College of Physicians of London, King's Fund Centre & National Asthma Campaign. Guidelines For Management Of Asthma In Adults: I: Chronic Persistent Asthma. *Br. Med. J.* **301,** 651–653 (1990).
7. Kim, S. Y. *et al.* Incidence and Risk Factors of Steroid-induced Diabetes in Patients with Respiratory Disease. *J Korean Med Sci* **26,** 264–267 (2011).
8. Suissa, S., Kezouh, A. & Ernst, P. Inhaled Corticosteroids and the Risks of Diabetes Onset and Progression. *AJM* **123,** 1001–1006 (2010).
9. Blackburn, D., Hux, J. & Mamdani, M. Quantification of the risk of corticosteroid-induced diabetes mellitus among the elderly. *J. Gen. Intern. Med.* **17,** 717–720 (2002).
10. Adinoff, A. D. & Hollister, J. R. Steroid-Induced Fractures and Bone Loss in Patients with Asthma. *N. Engl. J. Med.* **309,** 265–268 (1983).
11. Van Staa, T. P., Leufkens, H. G., Abenhaim, L., Zhang, B. & Cooper, C. Use of oral corticosteroids and risk of fractures. *J Bone Min. Res* (2000). doi:10.1359/jbmr.2000.15.6.993
12. Bloechliger, M. *et al.* Adverse events profile of oral corticosteroids among asthma patients in the UK: cohort study with a nested case- control analysis. *Respir. Res.* **19,** (2018).
13. Dawson, K. L. & Carter, E. R. A steroid-induced acute psychosis in a child with asthma. *Pediatr. Pulmonol.* **26,** 362–364 (1998).
14. Kayani, S. & Shannon, D. C. Adverse behavioral effects of treatment for acute exacerbation of asthma in children: A comparison of two doses of oral steroids. *Chest* **122,** 624–628 (2002).
15. Brown, E. S., Khan, D. A. & Nejtek, V. A. The psychiatric side effects of corticosteroids. *Ann. Allergy, Asthma Immunol.* **83,** 495–504 (1999).
16. Royal College of Physcians. *Why asthma still kills: The National Review of Asthma Deaths (NRAD)*. (2014).
17. Green, R. H., Brightling, C. E. & McKenna, S. Asthma exacerbations and eosinophil counts. A randomised controlled trial. *Lancet* **360,** 1715–21 (2002).
18. Buelo, A. *et al.* At-risk children with asthma (ARC): a systematic review. *Thorax* **01136,** 1–12 (2018).
19. Turner, M. O. *et al.* Risk factors for near-fatal asthma A case-control study in hospitalized patients with asthma. *Am. J. Respir. Crit. Care Med.* **157,** 1804–1809 (1998).
20. ten Brinke, A. *et al.* Risk factors of frequent exacerbations in difficult-to-treat asthma. *Eur. Respir. J.* (2005). doi:10.1183/09031936.05.00037905
21. Turner, S. W., Murray, C., Thomas, M., Burden, A. & Price, D. B. Applying UK real-world primary care data to predict asthma attacks in 3776 well-characterised children: a retrospective cohort study. *npj Prim. Care Respir. Med.* **28,** 28 (2018).
22. Loymans, R. J. B. *et al.* Identifying patients at risk for severe exacerbations of asthma:

For peer review only

development and external validation of a multivariable prediction model. *Thorax* **71,** 838–846 (2016).

23. Robroeks, C. M. H. H. T. *et al.* Prediction of asthma exacerbations in children: Results of a one-year prospective study. *Clin. Exp. Allergy* **42,** 792–798 (2012).
24. Haselkorn, T. *et al.* Recent asthma exacerbations predict future exacerbations in children with severe or difficult-to-treat asthma. *J. Allergy Clin. Immunol.* **124,** 921–927 (2009).
25. Bateman, E. D. *et al.* Development and validation of a novel risk score for asthma exacerbations: The risk score for exacerbations. *J. Allergy Clin. Immunol.* **135,** 1457–1464e4 (2015).
26. Boslev, C., Md, B., Suppli, C. & Dmsc, U. M. Asthma and Adherence to Inhaled Corticosteroids: Current Status and Future Perspectives. *Respir Care* **60,** 455–468 (2015).
27. Guedes, A. *et al.* Risk factors for death in patients with severe asthma. *J Bras Pneumol* **40,** 364–372 (2014).
28. Engelkes, M., Janssens, H. M., De Jongste, J. C., Sturkenboom, M. C. J. M. & Verhamme, K. M. C. Medication adherence and the risk of severe asthma exacerbations: a systematic review. *Eur Respir J* **45,** 396–407 (2015).
29. Papi, A. *et al.* Relationship of Inhaled Corticosteroid Adherence to Asthma Exacerbations in Patients with Moderate-to-Severe Asthma. *J. Allergy Clin. Immunol. Pract.* (2018). doi:10.1016/j.jaip.2018.03.008
30. McCarville, M., Sohn, M. W., Oh, E., Weiss, K. & Gupta, R. Environmental tobacco smoke and asthma exacerbations and severity: The difference between measured and reported exposure. *Arch. Dis. Child.* **98,** 510–514 (2013).
31. Marquette, C. H. *et al.* Long-term Prognosis of Near-Fatal Asthma. (1991).
32. Price, D. *et al.* Predicting frequent asthma exacerbations using blood eosinophil count and other patient data routinely available in clinical practice. *J. Asthma Allergy* 1 (2016). doi:10.2147/JAA.S97973
33. Black, M. H., Zhou, H., Takayanagi, M., Jacobsen, S. J. & Koebnick, C. Increased asthma risk and asthma-related health care complications associated with childhood obesity. *Am. J. Epidemiol.* **178,** 1120–1128 (2013).
34. Schatz, M. *et al.* Overweight/obesity and risk of seasonal asthma exacerbations. *J. Allergy Clin. Immunol. Pract.* **1,** 618–622 (2013).
35. Quinto, K. B. *et al.* The association of obesity and asthma severity and control in children. *J. Allergy Clin. Immunol.* **128,** 964–969 (2011).
36. Stingone, J. A., Ramirez, O. F., Svensson, K. & Claudio, L. Prevalence, demographics, and health outcomes of comorbid asthma and overweight in urban children. *J. Asthma* **48,** 876–885 (2011).
37. Sarpong, S. B. & Karrison, T. Sensitization to indoor allergens and the risk for asthma hospitalization in children. *Ann. Allergy, Asthma Immunol.* **79,** 455–459 (1997).
38. Stingone, J. A. & Claudio, L. Disparities in the use of urgent health care services among asthmatic children. *Ann. Allergy, Asthma Immunol.* **97,** 244–250 (2006).
39. Schatz, M., Cook, E. F., Joshua, A. & Petitti, D. Risk Factors for Asthma Hospitalizations in a Managed Care Organization: Development of a Clinical Prediction Rule. *Am. J. Manag. Care* **9,** 538–547 (2003).
40. Rosas-Salazar, C. *et al.* Parental numeracy and asthma exacerbations in Puerto Rican children. *Chest* **144,** 92–98 (2013).
41. Bossios, A. & Papadopoulos, N. *Viruses and asthma exacerbations*. **3,** (2006).
42. Leung, D. Y. M., Ledford, D. K., Jackson, D. J., Johnston, S. L. & London, P. Clinical reviews in allergy and immunology The role of viruses in acute exacerbations of

asthma. *J. Allergy Clin. Immunol.* **125,** 1178–1187 (2010).
43. Busse, W. W., Lemanske, R. F. & Gern, J. E. The Role of Viral Respiratory Infections in Asthma and Asthma Exacerbations NIH Public Access. *Lancet* **376,** 826–834 (2010).
44. King, G., Zeng, L. & King, G. Logistic Regression in Rare Events Data. *Polit. Anal.* **9,** 137–163 (2001).
45. Lieu, T. A., Quesenberry, C. P., Sorel, M. E., Mendoza, G. R. & Leong, A. B. Computer-based models to identify high-risk children with asthma. *Am J Respir Crit Care Med* (1998). doi:10.1164/ajrccm.157.4.9708124
46. Smith, J. R. *et al.* The at-risk registers in severe asthma (ARRISA) study: A cluster-randomised controlled trial examining effectiveness and costs in primary care. *Thorax* **67,** 1052–1060 (2012).
47. Loymans, R. J. B. *et al.* Exacerbations in Adults with Asthma: A Systematic Review and External Validation of Prediction Models. *J. Allergy Clin. Immunol. Pract.* (2018). doi:10.1016/j.jaip.2018.02.004
48. Van Vliet, D. *et al.* Prediction of asthma exacerbations in children by innovative exhaled inflammatory markers: Results of a longitudinal study. *PLoS One* **10,** 1–15 (2015).
49. Hallit, S. *et al.* Development of an asthma risk factors scale (ARFS) for risk assessment asthma screening in children. *Pediatr. Neonatol.* (2018). doi:10.1016/j.pedneo.2018.05.009
50. Forno, E. *et al.* Risk factors and predictive clinical scores for asthma exacerbations in childhood. *Chest* **138,** 1156–1165 (2010).
51. Finkelstein, J. & Jeong, I. cheol. Machine learning approaches to personalize early prediction of asthma exacerbations. *Ann. N. Y. Acad. Sci.* **1387,** 153–165 (2017).
52. Soyiri, I. N. *et al.* Improving predictive asthma algorithms with modelled environment data for Scotland: an observational cohort study protocol. *BMJ Open* **8,** e23289 (2018).
53. Simpson, C. R. *et al.* Evaluating the effectiveness, impact and safety of live attenuated and seasonal inactivated influenza vaccination: protocol for the Seasonal Influenza Vaccination Effectiveness II (SIVE II) study. doi:10.1136/bmjopen-2016
54. Simpson, C. R. *et al.* Seasonal Influenza Vaccination Effectiveness II (SIVE II): an observational study to evaluate live attenuated and trivalent inactivated influenza vaccination effectiveness, public health impact and safety – 2010/11 to 2015/16 seasons. *Heal. Technol Assess.* (in press)
55. Reddel, H. K. *et al.* An official American Thoracic Society/European Respiratory Society statement: Asthma control and exacerbations - Standardizing endpoints for clinical asthma trials and clinical practice. *Am. J. Respir. Crit. Care Med.* **180,** 59–99 (2009).
56. Scottish Government National Statistics Publications. *Introducing The Scottish Index of Multiple Deprivation 2016*. (2016).
57. Scottish Government. *Scottish Government Urban Rural Classification 2016*.
58. Scottish Government. *Review of Nomenclature of Units for Territorial Statistics (NUTS) Boundaries*. (2016).
59. British Thoracic Society. *British Guideline on the Management of Asthma: Quick Reference Guide*. *Scottish Intercollegiate Guidelines Network* (2016). doi:10.1136/thx.2008.097741
60. Lewis, J. D. & Brensinger, C. Agreement between GPRD smoking data: A survey of general practitioners and a population-based survey. *Pharmacoepidemiol. Drug Saf.* **13,** 437–441 (2004).
61. Marston, L. *et al.* Issues in multiple imputation of missing data for large general

For peer review only

practice clinical databases. *Pharmacoepidemiol. Drug Saf.* **19,** 618–626 (2010).
62. Deyo, R. A., Cherkin, D. C. & Ciol, M. A. Adapting a clinical comorbidity index for use with ICD-9-CM administrative databases. *J. Clin. Epidemiol.* **45,** 613–619 (1992).
63. Charlson, M. E., Pompei, P., Ales, K. L. & MacKenzie, R. A new method of classifying prognostic in longitudinal studies: development and validation. *Journal of Chronic Diseases* **40,** 373–383 (1987).
64. Blakey, J. D. *et al.* Identifying Risk of Future Asthma Attacks Using UK Medical Record Data: A Respiratory Effectiveness Group Initiative. *J. Allergy Clin. Immunol. Pract.* **5,** 1015–1024.e8 (2017).
65. He, H. & Garcia, E. A. Learning from Imbalanced Data. *IEEE Trans. Knowl. DATA Eng.* **21,** 1263–1284 (2009).
66. Chawla, N. V., Bowyer, K. W., Hall, L. O. & Kegelmeyer, W. P. SMOTE: Synthetic Minority Over-sampling Technique. *J. Artif. Intell. Res.* **16,** 321–357 (2002).
67. Kiefer, J. Sequential minimax search for a maximum. *Proc. Am. Math. Soc.* (1953). doi:10.2307/2032161
68. Chicco, D. Ten quick tips for machine learning in computational biology. *BioData Min.* **10,** 1–17 (2017).
69. Hripcsak, G. & Rothschild, A. S. Agreement, the F-measure, and reliability in information retrieval. *J. Am. Med. Informatics Assoc.* **12,** 296–298 (2005).
70. Collins, G. S., Reitsma, J. B., Altman, D. G. & Moons, K. G. Transparent reporting of a multivariable prediction model for individual prognosis or diagnosis (TRIPOD): the TRIPOD Statement. *BMC Med.* (2015). doi:10.1186/s12916-014-0241-z
71. Nicholls, S. G. *et al.* The REporting of studies Conducted using Observational Routinely-collected health Data (RECORD) statement: Methods for arriving at consensus and developing reporting guidelines. *PLoS One* **10,** 1–23 (2015).
72. Majka, M. CRAN: Package 'naivebayes' (version 0.9.2). *https://cran.r-project.org/web/packages/naivebayes/naivebayes.pdf* (2018).
73. Meyer, D. *et al.* CRAN: Package 'e1071' (version 1.7-0). *https://cran.r-project.org/web/packages/e1071/e1071.pdf* (2018).
74. Chang, C.-C. & Lin, C.-C. LIBSVM : a library for support vector machines. *ACM Trans. Intell. Syst. Technol.* **2,** (2011).
75. Breiman, L., Cutler, A., Liaw, A. & Wiener, M. CRAN: Package 'randomForest' (version 4.6-14). *https://cran.r-project.org/web/packages/randomForest/randomForest.pdf* (2018).
76. Chen, T. *et al.* CRAN: package 'xgboost' (version 0.71.2). *https://cran.r-project.org/web/packages/xgboost/xgboost.pdf* (2018).

For peer review only

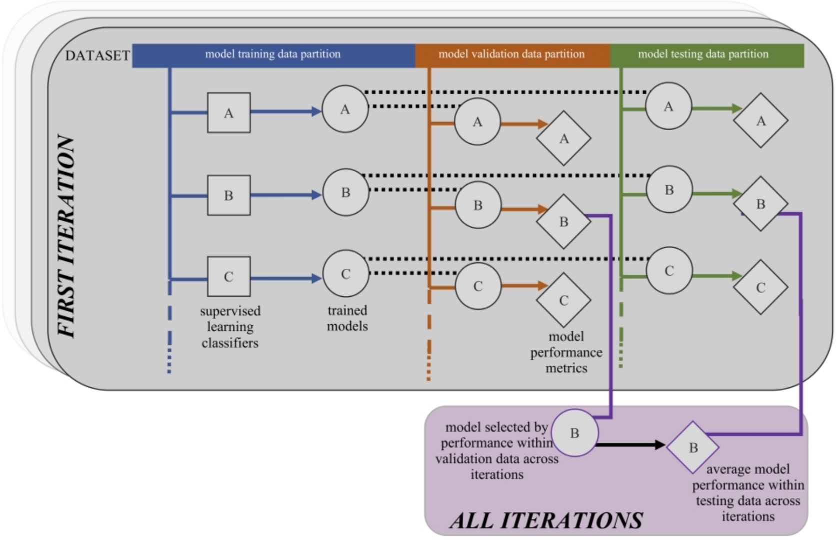

Process of selecting the highest performing model from the validation data, and the average performance of this model across iterations in the testing dataset. In the foreground we have the first iteration. We will use 100 iterations for statistical confidence, randomly permuting the data into training, validation, and testing subsets in each iteration

297x209mm (300 x 300 DPI)

Appendix A – Data harmonisation plan

Variable	Derivation dataset (LHS) format	External (unseen; SIVE II) dataset format	Harmonised format
Sex	Character – "M", "F" and "I" (less than 0.001% of records)	Character – "M", "F"	Character – "M", "F" and "I"
Birthday	Age (integer) at data extraction date (31st March 2018) or deduction date (indicated)	YYYY-MM-DD date format, all days set to 01 (true day redacted)	Age on March 31st, 2015 (approximate)
Scottish Index of Multiple Deprivation	Quintiles, 2012 and 2009 values	Deciles, 2012 values	Quintiles, 2012 values
Scottish Government Urban Rural Classification Scale	6-fold scale, from (1) Large Urban Areas to (6) Remote Rural Areas	8-fold scale, from (1) Large Urban Areas to (8) Very Remote Rural Areas	6-fold scale, from (1) Large Urban Areas to (6) Remote Rural Areas, 8-fold scale recoded as follows:<br>1 > 1<br>2 > 2<br>3 > 3<br>4, 5 > 4<br>6 > 5<br>7,8 > 6
Cause of death	ICD10 coded primary field, and 10 secondary cause fields	ICD10 coded primary field, and 10 secondary cause fields	*Aligned*
A&E cause of presentation	Presenting complaint free text field and 3 ICD10 coded disease fields	Presenting complaint free text field and 3 ICD10 coded disease fields	*Aligned*
Primary care records	Read Codes (version 2)	Read Codes (version 2)	*Aligned*
Primary care prescriptions	Standardised [a] text drug name and dose fields	Standardised [a] text drug name and dose fields	*Aligned*
Hospital inpatient admission records	N/A	ICD10 coded primary field, and 5 secondary cause fields	*Omitted as alignment not possible*
Event Date	Standardised date format	Standardised date format	*Aligned*

[a] Auto-fill assisted free text field

For peer review only

Appendix B – Machine Learning classifier hyperparameters

Naïve Bayes Classifier
Implemented using the r function *naivebayes*, from the package of the same name [72].
No hyperparameters.

Support Vector Machine
Implemented using the r function *svm*, from the package *e1071* [73] *which builds upon the LIBSVM package* [74], using a radial basis kernel function.

- *GAMMA* = Radial basis kernel function gamma parameter, corresponding to the kernel bandwidth (default 1/k): $2^{(-5:10)}$
- *COST* = Cost of constraints violation, i.e. samples penalised when crossing the boundary (default 1): $2^{(-5:10)}$

Ensemble: Bagging
Bagging methods learn from multiple models which are staged in parallel.
Random Forests
Implemented using the r function *randomForest*, from the package of the same name [75].

- *NTREE* = Number of trees to grow (default 500): 500, 750, 1000
- *MTRY* = Number of variables randomly sampled as candidates at each split (default square root of the number of predictors; k): floor($0.5 * \sqrt{k}$), floor($\sqrt{k}$), floor($2 * \sqrt{k}$) – in which floor represents the rounded-down integer value.

Ensemble: Boosting
Learning from multiple models which are staged *sequentially*, usually tree-based, constructed from different subsamples of the training dataset.
Extreme Gradient Boosting
Implemented using the r package *xgboost* [76], with 10-fold cross validation, repeated 3 times.

- NROUNDS = maximum number of iterations (default 100): 50,100
- MAXDEPTH = Maximum depth of each tree (default = 6): (1:5)^2
- ETA = step size of each boosting step (default = 0.3): 0.25, 0.5, 1

Ensemble: Stacking
Combining models from different classifiers, with an over-arching supervisor model which determines the best way to use all sources of information for prediction. The base set of weak learners will comprise all aforementioned model and hyperparameter combinations, and the meta-learner (random forest with 500 trees and mtry = floor($0.5 * \sqrt{k}$)) will use all weak learners with a validation set performance in the top 50%.

For peer review only

TRIPOD Checklist: Prediction Model Development and Validation

Section/Topic	Item		Checklist Item	Page
**Title and abstract**				
Title	1	D;V	Identify the study as developing and/or validating a multivariable prediction model, the target population, and the outcome to be predicted.	1* (* main paper will include validation)
Abstract	2	D;V	Provide a summary of objectives, study design, setting, participants, sample size, predictors, outcome, statistical analysis, results, and conclusions.	2** (** more thorough in main paper)
**Introduction**				
Background and objectives	3a	D;V	Explain the medical context (including whether diagnostic or prognostic) and rationale for developing or validating the multivariable prediction model, including references to existing models.	4
	3b	D;V	Specify the objectives, including whether the study describes the development or validation of the model or both.	5
**Methods**				
Source of data	4a	D;V	Describe the study design or source of data (e.g., randomized trial, cohort, or registry data), separately for the development and validation data sets, if applicable.	5
	4b	D;V	Specify the key study dates, including start of accrual; end of accrual; and, if applicable, end of follow-up.	6
Participants	5a	D;V	Specify key elements of the study setting (e.g., primary care, secondary care, general population) including number and location of centres.	5
	5b	D;V	Describe eligibility criteria for participants.	5-6
	5c	D;V	Give details of treatments received, if relevant.	-
Outcome	6a	D;V	Clearly define the outcome that is predicted by the prediction model, including how and when assessed.	6
	6b	D;V	Report any actions to blind assessment of the outcome to be predicted.	-
Predictors	7a	D;V	Clearly define all predictors used in developing or validating the multivariable prediction model, including how and when they were measured.	7-8**
	7b	D;V	Report any actions to blind assessment of predictors for the outcome and other predictors.	-
Sample size	8	D;V	Explain how the study size was arrived at.	6
Missing data	9	D;V	Describe how missing data were handled (e.g., complete-case analysis, single imputation, multiple imputation) with details of any imputation method.	7-8**
Statistical analysis methods	10a	D	Describe how predictors were handled in the analyses.	8-9
	10b	D	Specify type of model, all model-building procedures (including any predictor selection), and method for internal validation.	8-9
	10c	V	For validation, describe how the predictions were calculated.	8-9
	10d	D;V	Specify all measures used to assess model performance and, if relevant, to compare multiple models.	8-9
	10e	V	Describe any model updating (e.g., recalibration) arising from the validation, if done.	-**
Risk groups	11	D;V	Provide details on how risk groups were created, if done.	-
Development vs. validation	12	V	For validation, identify any differences from the development data in setting, eligibility criteria, outcome, and predictors.	9**
**Results**				
Participants	13a	D;V	Describe the flow of participants through the study, including the number of participants with and without the outcome and, if applicable, a summary of the follow-up time. A diagram may be helpful.	Protocol Paper
	13b	D;V	Describe the characteristics of the participants (basic demographics, clinical features, available predictors), including the number of participants with missing data for predictors and outcome.	Protocol Paper
	13c	V	For validation, show a comparison with the development data of the distribution of important variables (demographics, predictors and outcome).	Protocol Paper
Model development	14a	D	Specify the number of participants and outcome events in each analysis.	Protocol Paper
	14b	D	If done, report the unadjusted association between each candidate predictor and outcome.	Protocol Paper
Model specification	15a	D	Present the full prediction model to allow predictions for individuals (i.e., all regression coefficients, and model intercept or baseline survival at a given time point).	Protocol Paper
	15b	D	Explain how to the use the prediction model.	Protocol Paper
Model performance	16	D;V	Report performance measures (with CIs) for the prediction model.	Protocol Paper
Model-updating	17	V	If done, report the results from any model updating (i.e., model specification, model performance).	Protocol Paper
**Discussion**				
Limitations	18	D;V	Discuss any limitations of the study (such as nonrepresentative sample, few events per predictor, missing data).	Protocol Paper
Interpretation	19a	V	For validation, discuss the results with reference to performance in the development data, and any other validation data.	Protocol Paper
	19b	D;V	Give an overall interpretation of the results, considering objectives, limitations, results from similar studies, and other relevant evidence.	Protocol Paper
Implications	20	D;V	Discuss the potential clinical use of the model and implications for future research.	Protocol Paper
**Other information**				
Supplementary information	21	D;V	Provide information about the availability of supplementary resources, such as study protocol, Web calculator, and data sets.	Protocol Paper
Funding	22	D;V	Give the source of funding and the role of the funders for the present study.	Abstract

For peer review only - http://bmjopen.bmj.com/site/about/guidelines.xhtml

TRIPOD Checklist: Prediction Model Development and Validation

*Items relevant only to the development of a prediction model are denoted by D, items relating solely to a validation of a prediction model are denoted by V, and items relating to both are denoted D;V. We recommend using the TRIPOD Checklist in conjunction with the TRIPOD Explanation and Elaboration document.

For peer review only

BMJ Open

Predicting asthma attacks in primary care: protocol for developing a machine learning-based prediction model

Journal:	*BMJ Open*
Manuscript ID	bmjopen-2018-028375.R1
Article Type:	Protocol
Date Submitted by the Author:	02-Apr-2019

Complete List of Authors:	Tibble, Holly; University of Edinburgh School of Molecular Genetic and Population Health Sciences; Asthma UK Centre for Applied Research<br>Tsanas, Athanasios; University of Edinburgh; Asthma UK Centre for Applied Research<br>Horne, Elsie; University of Edinburgh; Asthma UK Centre for Applied Research<br>Horne, Robert; University College London; Asthma UK Centre for Applied Research<br>Mizani, Mehrdad; University of Edinbugh; Asthma UK Centre for Applied Research<br>Simpson, Colin; Victoria University of Wellington, School of Health; Asthma UK Centre for Applied Research<br>Sheikh, Aziz; University of Edinburgh, Division of Community Health Sciences; Asthma UK Centre for Applied Research

**Primary Subject Heading**:	Health informatics
Secondary Subject Heading:	Respiratory medicine, Public health
Keywords:	Asthma < THORACIC MEDICINE, PRIMARY CARE, Asthma Attacks, Machine Learning, Prediction

SCHOLARONE™
Manuscripts

Predicting asthma attacks in primary care: protocol for developing a machine learning-based prediction model

Word Count 3105/4000

Authors:
Holly Tibble [1,2] (corresponding author), Athanasios Tsanas [1,2], Elsie Horne [1,2], Robert Horne [2,3], Mehrdad A. Mizani [1,2], Colin R. Simpson [4,2], Aziz Sheikh [1,2]

1. Usher Institute of Population Health Sciences and Informatics, Edinburgh Medical School, College of Medicine and Veterinary Medicine, University of Edinburgh – Teviot Place, Edinburgh, United Kingdom
2. Asthma UK Centre for Applied Research, Usher Institute of Population Health Sciences and Informatics, Centre for Medical Informatics, The University of Edinburgh, Edinburgh, UK
3. Centre for Behavioural Medicine, Department for Practice and Policy, UCL School of Pharmacy, University College London – Mezzanine Floor, London, United Kingdom
4. School of Health, Faculty of Health, Victoria University of Wellington, Wellington, New Zealand

Author's Email Addresses:
Holly.tibble@ed.ac.uk
Athanasios.Tsanas@ed.ac.uk
Elsie.Horne@ed.ac.uk
r.horne@ucl.ac.uk
Mehrdad.Mizani@ed.ac.uk
c.simpson@ed.ac.uk
Aziz.Sheikh@ed.ac.uk

Author Contributions:
HT and AT conceived and planned the analysis. HT and RH specified the medication adherence measures. HT, EH, CS, MM, and AS constructed the covariate (and associated Read Coding) lists for the model. HT wrote the first draft, with contributions from all authors. All authors (HT, AT, EH, RH, MM, CR, and AS) approved the final version and jointly take responsibility for the decision to submit this manuscript to be considered for publication.

Conflicts of Interest:
None to report

Keywords:
Asthma, Asthma Attacks, Primary Care, Machine Learning, Prediction

Acknowledgements:
The authors would like to thank Eleftheria Vasileiou, Amy Tilbrook, Mome Mukherjee and Jill Tibble for their contributions to the analysis plan, data management, and proof-reading of this manuscript, and the Asthma UK Centre for Applied Research (AUKCAR) Patient and Public Involvement group for their contribution to the analysis plan.

For peer review only

ABSTRACT

Introduction

Asthma is a long-term condition with rapid onset worsening of symptoms ('attacks') which can be unpredictable and may prove fatal. Models predicting asthma attacks require high sensitivity to minimise mortality risk, and high specificity to avoid unnecessary prescribing of preventative medications that carry an associated risk of adverse events. We aim to create a risk score to predict asthma attacks in primary care using a statistical learning approach trained on routinely collected electronic health record (EHR) data.

Methods and Analysis

We will employ machine learning classifiers (naïve Bayes, support vector machines, and random forests) to create an asthma attack risk prediction model, using the Asthma Learning Health System (ALHS) study patient registry comprising 500,000 individuals from across 75 Scottish general practices, with linked longitudinal primary care prescribing records, primary care Read codes, accident and emergency records, hospital admissions and deaths. Models will be compared on a partition of the dataset reserved for validation, and the final model will be tested in both an unseen partition of the derivation dataset and in an external dataset from the Seasonal Influenza Vaccination Effectiveness II (SIVE II) study.

Ethics and Dissemination

Permissions for the ALHS project were obtained from the South East Scotland Research Ethics Committee 02 [16/SS/0130] and the Public Benefit and Privacy Panel for Health and Social Care [1516-0489]. Permissions for the SIVE II project were obtained from the Privacy Advisory Committee (National Services NHS Scotland) [68/14] and the National Research Ethics Committee West Midlands - Edgbaston [15/WM/0035]. The subsequent research paper will be submitted for publication to a peer-reviewed journal and code scripts used for all components of the data cleaning, compiling, and analysis will be made available in the open source GitHub website (https://github.com/hollytibble).

For peer review only

ARTICLE SUMMARY

Strengths and limitations of this study

- This analysis is based on a large, representative dataset comprising over 500,000 individuals recruited from 75 general practices from across Scotland
- We will employ novel applications of established machine learning and training data enrichment methodologies
- The prediction model we develop will be tested in unseen large external dataset, namely the SIVE II dataset
- This derivation and validation work will be undertaken in NHS Scotland; there will therefore be a need for further validation work in other UK nations and international contexts.

FUNDING STATEMENT

HT is supported by College of Medicine and Veterinary Medicine PhD (eHERC/Farr Institute) Studentships from The University of Edinburgh. EH is supported by a Medical Research Council PhD Studentship (eHERC/Farr). MAM's Newton International Fellowship is awarded by the Academy of Medical Sciences and Newton Fund. This work is carried out with the support of the Asthma UK Centre for Applied Research [AUK-AC-2012-01] and Health Data Research UK, an initiative funded by UK Research and Innovation Councils, National Institute for Health Research (England) and the UK devolved administrations, and leading medical research charities. The ALHS dataset was created with funding from the National Environment Research Council [NE/P011012/1]. The SIVE II dataset was created with funding from the National Institute for Health Research (NIHR) Health Technology Assessment programme [13/34/14] - the views and opinions expressed therein are those of the authors and do not necessarily reflect those of the Health Technology Assessment programme, NIHR, NHS or the Department of Health.

For peer review only

INTRODUCTION

Asthma is a long-term lung disease characterised by inflammation of the airways, which may manifest as episodic wheezing, chest tightness, coughing and shortness of breath. An asthma attack is the sudden worsening of symptoms, which may prove fatal [1]. In 2017, asthma was estimated to affect 235 million people worldwide [2]. In 2015 alone, 1,434 people died from asthma attacks in the United Kingdom (UK) – a rate of 2.21 deaths per 100,000 person-years [3]. Asthma attack incidence is reported to be between 0.01 and 0.78 events per person-year, depending on the definition of attacks, and the population (e.g. primary care, secondary care) [4–6].

Asthma therapy typically follows a fairly linear path – beginning with a short-acting bronchodilator in those without persistent asthma symptoms, and adding preventative treatments and long-acting bronchodilators in those with more persistent asthma symptoms [7,8]. Those with persistent troublesome symptoms and/or considered to be at very high risk may be prescribed biologicals and/or oral steroids [9]. Oral steroids are often considered a last resort, due to their undesirable safety profile including increased risk of diabetes [10–12], osteoporosis [13–15], and affective and psychotic disorders [15–18].

It follows that the determination of those at high risk for asthma attacks is crucial in order to prevent attacks and minimise the risk of unnecessary side-effects. Furthermore, the 2014 National Review of Asthma Deaths found that 45% of asthma deaths in the study year died without requesting medical help, or before help could be provided [5]. Increased awareness of the risk could prevent those with asthma from delay in seeking medical care and preventing fatality.

While it might seem intuitive that those with the most severe daily symptoms exhibit greater risk of severe morbidity and mortality, research suggests that these symptoms may be a suboptimal clinical marker of asthma attack risk [19]. Indeed, some people with asthma are more prone to asthma attacks than others, with past asthma attack history being the strongest risk factor for future asthma attacks [20–23]. Other commonly identified risk factors for asthma attacks include poor asthma control [24–27] (often a result of poor adherence to preventative therapy [28–31]), smoking [24,27,32–34], history of hospital admission [21,24], history of oral steroid use [24], obesity [27,34–38], socio-economic factors such as access to medicines [39,40] socioeconomic status [41,42], and viral respiratory infections [43–45].

Despite the identification of many risk factors, identifying high risk individuals has proven a challenging task. Logistic regression, the most commonly used statistical method for event prediction, is known to predict outcomes poorly when there is *class imbalance* (event and no event) [46], and we expect the problem investigated in this study assessing asthma attacks will be highly imbalanced. For example, a model could predict that a very rare event would never occur, and it would be correct in the vast majority of cases. As such, most prediction models report high *specificity* (correctly predicting low attack risk to those who did not have attacks), but low *sensitivity* (correctly predicting high risk in those who did go on to have attacks) [4,24,41,47–51], which results in less reliable risk prediction for patients at high risk.

In a recent study by Finkelstein and Jeong [52], sensitivity (and specificity) in excess of 75% was achieved for all classifiers (Adaptive Bayesian network, Naïve Bayes classifier, and Support Vector Machine) predicting asthma attacks a week in advance using a sample of just over 7000 records of home tele-monitoring data. They found substantial improvements in model

For peer review only

sensitivity using *training enrichment* methods; pre-processing the training data to improve the performance in the testing data – for example, by increasing the prevalence of the rare outcome in the training data to balance the classes.

RESEARCH AIM

We aim to create a personalised risk assessment tool to assist primary care clinicians in predicting asthma attacks over a period of 1, 4, 12, 26, and 52 weeks, employing machine learning methodologies such as naïve Bayes classifiers, random forests, and support vector machines, as well as ensemble algorithms. The model will build on previous research [4,24,41,47–52] to improve the sensitivity of our event prediction, without unduly compromising the specificity. This is crucial in order to reduce steroid prescribing and diminish the long-term effects of high steroid use over a life time, which have adverse effects [10–18], and reduce patient anxiety when risk of an asthma attack is low.

Primary care consultations provide the opportunity for patients and clinicians to assess changes to asthma attack risk, which can be used to promote patients to seek emergency care if there is a significant deterioration in their symptoms, and to promote risk-reducing lifestyle choices.

METHODS

Data Sources and Permissions

The derivation dataset used for training, validating, and testing the model will be the Asthma Learning Healthcare System (ALHS) dataset, created in order to develop and validate a prototype learning health system for asthma patients in Scotland [53]. The ALHS study aims to increase understanding of variation in asthma outcomes and create benchmarks for clinical practice in order to reduce sub-optimal care, by repurposing patient data to create a continuous loop of knowledge-generation, evidence-based clinical practice change, and change assessment. The study dataset contains patient demographics from the patient registry, primary care prescribing records, primary care encounters, Accident and Emergency (A&E) records, hospital inpatient admissions and deaths, linkable by an anonymised unique identifier. Datasets were extracted between November 2017 and August 2018 for the period January 2000 to December 2017, as shown in Table 1, along with the number of records and unique individuals before data cleaning.

In order to verify that the prediction model performance is not limited to the development dataset and that it generalizes well in new, unseen data presented to the classifier in the training process, we will evaluate its performance using an external cohort study dataset, the second Seasonal Influenza Vaccination Effectiveness (SIVE II) cohort study [54,55], which used a large national primary care (1.25 million individuals from 230 Scottish general practices) and laboratory-linked dataset to evaluate live attenuated and trivalent inactivated influenza vaccination effectiveness. The SIVE II dataset contains records from the same sources (primary and secondary care) and modalities (diagnosis and date) as the ALHS dataset (extraction and specification dates are shown in Table 2), and as such can be harmonised such that variables and value sets are aligned. In Appendix A, we detail the data harmonisation plan, that is, we list the key variables to be used in the following analyses, their format in each dataset (for example, whether age is pre-coded into 5-year bands) and the common denominator format that will be used in the analyses to ensure the highest degree of concordance during the validation stage.

For peer review only

Permissions for the ALHS project were obtained from the South East Scotland Research Ethics Committee 02 [16/SS/0130] and the Public Benefit and Privacy Panel for Health and Social Care [1516-0489]. Permissions for the SIVE II project were obtained from the Privacy Advisory Committee (National Services NHS Scotland) [68/14] and the National Research Ethics Committee West Midlands - Edgbaston [15/WM/0035].

Patient and Public Involvement

This analysis plan was constructed with the assistance of the Asthma UK Centre for Applied Research (AUKCAR) Patient and Public Involvement (PPI) group. The particular importance of avoiding a substantial decrease in specificity in order to gain higher sensitivity was a result of discussions within this group about the burden of side-effects from preventative treatment.

Inclusion Criteria

We will identify our study population as all adults (aged 18 and over) with asthma identified by clinical diagnoses (Read codes), without a chronic obstructive pulmonary disease (COPD) diagnosis, and with relevant prescribing records in primary care. Patients with missing sex or age information will be removed; this and any other patient exclusions from further analysis will be explicitly detailed.

All records from the derivation dataset (ALHS) will be left-censored at January 2009, in order to align with the primary care prescribing data, and right-censored at March 2017, in order to align with the mortality, primary care, and inpatient hospital admission records, are presented in Table 1. Similarly, records from the external dataset (SIVE II) will be left-censored at January 2003, in order to align with the primary care prescribing data, and right-censored at August 2016 to align with the A&E records, as shown in Table 2. There is a high probability that some individuals will have been recruited into both studies, and therefore those individuals will be flagged in the external testing dataset and removed from the study pool.

Table 1: Meta-data for Clinical Data sources in Derivation Dataset (ALHS)

Data Source	Number of Records	Number of Individuals	Extraction Date	Data Specification Date Range
Primary Care Prescribing a	4,709,231	47,095	October 2018	January 2009 – April 2017
Primary Care Encounters [a]	11,766,100	49,307	March 2018	January 2000 – November 2017
Accident & Emergency	1,831,789	500,321	November 2017	June 2007 – September 2017
Hospital Inpatient Admissions	1,668,957	342,838	August 2018	January 2000 – March 2017
Mortality	*NA*	91,758	May 2018	January 2000 – March 2017

a. Records available for subset of study population with asthma diagnosis only

For peer review only

Table 2: Meta-data for Clinical Data sources in External Dataset (SIVE II)

Data Source	Number of Records	Number of Individuals	Extraction Date	Data Specification Date Range
Primary Care Prescribing	29,360,448	1,073,377	May 2017	January 2003 – March 2017
Primary Care Encounters	31,878,423	1,887,957	May 2017	January 2000 [a] – March 2017
Accident & Emergency	4,116,561	1,247,314	April 2017	June 2007 - August 2016
Hospital Inpatient Admissions	3,549,174	794,937	April 2017	January 2000 - March 2017
Mortality	*NA*	215,466	April 2017	January 2000 - March 2017

[a] *Diagnosis codes entered in this period, but post-dated from 1940 onwards retained.*

Outcome Ascertainment

We will identify asthma attacks, defined by the American Thoracic Society/European Respiratory Society [56] as either a prescription of oral corticosteroids, an asthma-related A&E visit, or an asthma-related hospital admission. Additionally, deaths occurring with asthma as the primary cause will be labelled as asthma attacks. Instances of multiple attack indicators occurring within a 14-day period were coded as a single attack.

Patient Characteristics, Confounders, and Missing Data

Patient characteristics at baseline will be reported, and included as time-varying confounders in analyses. For all characteristics derived from Read codes, full code lists will be provided as supplementary materials.

Demographics: Age, sex, rurality, and social deprivation will be extracted from the primary care registry. Social deprivation is measured using quintiles of the Scottish Index of Multiple Deprivation (SIMD), a geographic measure derived using data on income, employment, education, health, access to services, crime and housing [57]. Rurality is defined using the Scottish Government Urban Rural Classification Scale (6-fold scale) [58]. While missing age and/or sex are exclusion criteria for the study sample, missingness for rurality and social deprivation will be coded as 'missing'.

Practice Location: Practice location will be included in order to account for clustering of patients by region. Location will be coded using the Nomenclature of Territorial Units for Statistics [59] (NUTS 3) codes, linked from the registered practice data zone (2001).

Asthma Severity: Asthma severity will be categorised using the British Thoracic Society's 2016 5-step treatment classification [60]. Severity will be considered time-dependent and will be determined using prescribing records at any change in regimen.

Smoking Status: Smoking status will be derived from primary care data, and presented as a 3-level variable, namely: current, former, and non-smoker, using the most recent smoking Read

For peer review only

code at any day. Smoking status will be considered time-dependent and determined using the most recent Read code records, and those with unknown smoking status will be coded as non-smokers [61,62].

Blood Eosinophil Count: Blood eosinophil count will be derived from primary care Read codes, and will be dichotomised at ≥400 cells per μL. Those with non-recorded blood eosinophil count will be coded as missing. Blood eosinophil count will be considered time-dependent and determined using the most recent Read code record.

Obesity: Obesity will be derived from Body Mass Index (BMI) or height and weight records in primary care data, and will be presented as a binary variable (BMI≥30). Those with unknown BMI will be coded as non-obese. Obesity will be considered time-dependent and determined using the most recent Read code record.

Comorbidity: Comorbidity will be defined by 17 dichotomous (unweighted) variables representing the diagnostic categories of the adapted Charlson Comorbidity Index [63,64]. Additionally, active diagnoses of rhinitis, eczema, gastroesophageal reflux disease (GERD), nasal polyps, and anaphylaxis will be recorded; all identified by Blakey et al. as contributing characteristics to increased asthma attack risk [65]. Comorbidities will be considered time-dependent and determined using all prior Read code records.

Previous Healthcare Usage: The number of repeat prescriptions of preventer medication, and the number of primary care asthma encounters (days on which at least one asthma related code was recorded) in the previous year will be derived from primary care prescribing and Read code records, respectively. Both will be considered time-dependent and determined using records from the previous calendar year.

Asthma Control: The mean Short-Acting Beta-2 Agonist (SABA) dose per day will be estimated retroactively by examining the dates between prescriptions. The most recent peak expiratory flow measurement at any time will be recorded (categorical, based on percentage of previous maximum) or coded as missing if that measurement was more than seven days ago. Adherence to preventer therapy will be approximated using the medication possession ratio [66], calculated from primary care prescribing records.

History of Asthma Attacks: Prior asthma attacks will be identified solely using primary care prescribing records and Read codes. This is because primary care practitioners will not be able to make use of secondary care records when utilising this risk score with patients. Both the prior number of attacks, and the time since the last attack, will be included as predictors and will be considered time-dependent and accurate at the weekly level.

Analysis Plan

The derivation dataset (ALHS) will be divided into three partitions: 60% for training, 20% for model comparison (validation), and 20% to assess performance (testing). In our training subset, the first partition, we will train machine learning models (classifiers) with varying hyperparameters, predicting asthma attack occurrence in the following 1, 4, 26, and 52 weeks. We will run 100 iterations for statistical confidence, each time randomly permuting samples prior to determining the three subsets. The *no free lunch theorem* in machine learning suggests there is no classifier (or more generically a machine learning tool) which will consistently outperform competing approaches across all settings [67]. Therefore, given that we do not know

For peer review only

a priori which classifier will work best in this application, we will apply naïve Bayes classifiers for benchmarking, and then employ more advanced state of the art principled supervised learning algorithmic tools such as support vector machines, random forests, and ensembles (classifier combinations), to investigate which algorithm leads to more accurate results.

A selection of *training enrichment* methods will be trialled, in order to assess how to best overcome poor performance as a result of low outcome prevalence. Typically, modelling rare events results in reduced sensitivity (the proportion of those who had attacks that were detected), so those predicted to be low-risk will have a high rate of asthma attacks. As such, this start of this process (the first 20 iterations of training each model) will be repeated five times, using:

1. the original analysis dataset,
2. original data with additional duplicates of the positive outcome records (a method known as *over-sampling* [68]),
3. original data, with a selection of the negative outcome records removed (*under-sampling* [68]),
4. original data with additional slightly modified duplicates of the positive outcome records, with a selection of the negative outcome records removed (*Synthetic minority over-sampling; SMOTE* [68,69]),
5. original data, using the outcome classification threshold to maximise the our primary metric - the Matthew's Correlation Coefficient (MCC) [70] – identified using golden-section search optimisation [71].

By assessing the average performance, by classification method class, in each set of iterations, we will determine which enrichment method is the most appropriate overall for the data, and continue accordingly.

In the validation partition, with all 100 iterations for the selected enrichment methods, we will identify the highest performing model as that with the highest mean MCC across iterations; in the event of a tie, the model with the highest iteration-minimum MCC will be selected.

Model testing will be conducted on the selected model (Figure 1) in the derivation testing partitions. Model calibration will be assessed by comparing observed rate of incidence by predicted risk, for the full population and by exhaustive population subgroups, including asthma severity, prior number of asthma attacks, age and smoking status (particularly useful to assess possible contamination by asthma-COPD overlap syndrome (ACOS)). We will also check the calibration between the predicted risk and the attack incidence, stratified by the source of the asthma attack record (in primary care, A&E presentation, or inpatient admission). Performance in the testing datasets will be assessed using the MCC, and the additional metrics of sensitivity, specificity, positive predictive value and negative predictive value, and the F_1 measure [72], along with information criteria such as the Bayesian Information Criterion (BIC) to obtain a trade-off between model complexity and accuracy. Confusion matrices (also known as contingency tables) will be made available as supplementary materials.

The derivation dataset will be re-used in its entirety to retrain the model based on the final classifier and hyperparameter selection. Model testing will then be conducted in the external dataset, which consists of data unseen in the model derivation, using this trained model. Distributions of predictors between the derivation and external datasets will be assessed (indirectly) to contextualise the generalisability findings. The aforementioned metrics will be reported.

For peer review only

Finally, we will re-train the model using the hyperparameter specifications from the best performing model, with a modified version of the derivation dataset which incorporates data extracted from secondary care records (such as A&E presentations for asthma attack not captured in primary care records) in the determination of the risk factors. This allows us to evaluate the added value of secondary care data linkage in the prediction of impending asthma attacks, and will be determined by the same metrics used for the primary model evaluation.

All analyses will be conducted in R (though the RStudio interface), and details on the functions, the hyperparameter within each classifier, and the ranges assessed herein, are provided in Appendix B.

[[INSERT FIGURE 1 HERE]]

Figure 1: Process of selecting the highest performing model from the validation data, and the average performance of this model across iterations in the testing dataset. In the foreground we have the first iteration. We will use 100 iterations for statistical confidence, randomly permuting the data into training, validation, and testing subsets in each iteration

Ethics and Dissemination

All authors with data access have completed the Safe Users of Research data Environment (SURE) training, provided by the Administrative Data Research Network (ADRN). All analysis will be conducted in concordance with the National Services Scotland Electronic Data Research and Innovation Service (eDRIS) user agreement. This study protocol will be registered with the European Union electronic Register of Post-Authorisation Studies (EU PAS Register) as a non-interventional post-authorisation study (PAS) before any data analysis is initiated.

The subsequent research paper will be submitted for publication in a peer-reviewed journal and will be written in accordance with TRIPOD: *transparent reporting of a multivariable prediction model for individual prognosis or diagnosis* [73] and RECORD: *reporting of studies conducted using observational routinely-collected health data* [74] guidelines. Code scripts used for all components of the data cleaning, compiling, and analysis will be made available in the open source GitHub website at https://github.com/hollytibble.

A lay summary of this protocol paper, and the subsequent research results paper, will be made available online (via an open source platform) in order to heighten the impact and accessibility of this work.

Data statement

The derivation and external datasets used in this study are accessible via the eDRIS secure platform under the project numbers 1516-0160 and 1516–0489, respectively.

Conclusions

This project will further advance asthma attack risk prediction modelling and will inform on the future direction of routine data linkage in Scotland, which is likely to have additional benefits for other health systems in the United Kingdom and internationally.

For peer review only

References

1. Bush, A. & Griffiths, C. Improving treatment of asthma attacks in children. *BMJ (Online)* (2017). doi:10.1136/bmj.j5763
2. World Health Organisation. *Asthma Fact Sheet (2017)*. *World Health Organisation Fact Sheets* (World Health Organization, 2017).
3. Asthma UK. *UK asthma death rates among worst in Europe*. (2017).
4. Loymans, R. J. B. *et al.* Exacerbations in Adults with Asthma: A Systematic Review and External Validation of Prediction Models. *J. Allergy Clin. Immunol. Pract.* (2018). doi:10.1016/j.jaip.2018.02.004
5. Royal College of Physcians. *Why asthma still kills: The National Review of Asthma Deaths (NRAD)*. (2014).
6. Mukherjee, M., Nwaru, B. I., Soyiri, I., Grant, I. & Sheikh, A. High health gain patients with asthma: a cross-sectional study analysing national Scottish data sets. *Prim. Care Respir. Med.* **28,** 27 (2018).
7. British Thoracic Society & Scottish Intercollegiate Guidelines Network. British guideline on the management of asthma. *SIGN Guidel.* (2014). doi:10.1136/thx.2008.097741
8. Currie, G. P., Douglas, J. G. & Heaney, L. G. Difficult to treat asthma in adults. *BMJ* **338,** b494 (2009).
9. British Thoracic Society, Research Unit of the Royal College of Physicians of London, King's Fund Centre & National Asthma Campaign. Guidelines For Management Of Asthma In Adults: I: Chronic Persistent Asthma. *Br. Med. J.* **301,** 651–653 (1990).
10. Kim, S. Y. *et al.* Incidence and Risk Factors of Steroid-induced Diabetes in Patients with Respiratory Disease. *J Korean Med Sci* **26,** 264–267 (2011).
11. Suissa, S., Kezouh, A. & Ernst, P. Inhaled Corticosteroids and the Risks of Diabetes Onset and Progression. *AJM* **123,** 1001–1006 (2010).
12. Blackburn, D., Hux, J. & Mamdani, M. Quantification of the risk of corticosteroid-induced diabetes mellitus among the elderly. *J. Gen. Intern. Med.* **17,** 717–720 (2002).
13. Adinoff, A. D. & Hollister, J. R. Steroid-Induced Fractures and Bone Loss in Patients with Asthma. *N. Engl. J. Med.* **309,** 265–268 (1983).
14. Van Staa, T. P., Leufkens, H. G., Abenhaim, L., Zhang, B. & Cooper, C. Use of oral corticosteroids and risk of fractures. *J Bone Min. Res* (2000). doi:10.1359/jbmr.2000.15.6.993
15. Bloechliger, M. *et al.* Adverse events profile of oral corticosteroids among asthma patients in the UK: cohort study with a nested case- control analysis. *Respir. Res.* **19,** (2018).
16. Dawson, K. L. & Carter, E. R. A steroid-induced acute psychosis in a child with asthma. *Pediatr. Pulmonol.* **26,** 362–364 (1998).
17. Kayani, S. & Shannon, D. C. Adverse behavioral effects of treatment for acute exacerbation of asthma in children: A comparison of two doses of oral steroids. *Chest* **122,** 624–628 (2002).
18. Brown, E. S., Khan, D. A. & Nejtek, V. A. The psychiatric side effects of corticosteroids. *Ann. Allergy, Asthma Immunol.* **83,** 495–504 (1999).
19. Green, R. H., Brightling, C. E. & McKenna, S. Asthma exacerbations and eosinophil counts. A randomised controlled trial. *Lancet* **360,** 1715–21 (2002).
20. Buelo, A. *et al.* At-risk children with asthma (ARC): a systematic review. *Thorax* **01136,** 1–12 (2018).
21. Turner, M. O. *et al.* Risk factors for near-fatal asthma A case-control study in hospitalized patients with asthma. *Am. J. Respir. Crit. Care Med.* **157,** 1804–1809 (1998).

For peer review only

22. ten Brinke, A. *et al.* Risk factors of frequent exacerbations in difficult-to-treat asthma. *Eur. Respir. J.* (2005). doi:10.1183/09031936.05.00037905
23. Turner, S. W., Murray, C., Thomas, M., Burden, A. & Price, D. B. Applying UK real-world primary care data to predict asthma attacks in 3776 well-characterised children: a retrospective cohort study. *npj Prim. Care Respir. Med.* **28,** 28 (2018).
24. Loymans, R. J. B. *et al.* Identifying patients at risk for severe exacerbations of asthma: development and external validation of a multivariable prediction model. *Thorax* **71,** 838–846 (2016).
25. Robroeks, C. M. H. H. T. *et al.* Prediction of asthma exacerbations in children: Results of a one-year prospective study. *Clin. Exp. Allergy* **42,** 792–798 (2012).
26. Haselkorn, T. *et al.* Recent asthma exacerbations predict future exacerbations in children with severe or difficult-to-treat asthma. *J. Allergy Clin. Immunol.* **124,** 921–927 (2009).
27. Bateman, E. D. *et al.* Development and validation of a novel risk score for asthma exacerbations: The risk score for exacerbations. *J. Allergy Clin. Immunol.* **135,** 1457–1464e4 (2015).
28. Boslev, C., Md, B., Suppli, C. & Dmsc, U. M. Asthma and Adherence to Inhaled Corticosteroids: Current Status and Future Perspectives. *Respir Care* **60,** 455–468 (2015).
29. Guedes, A. *et al.* Risk factors for death in patients with severe asthma. *J Bras Pneumol* **40,** 364–372 (2014).
30. Engelkes, M., Janssens, H. M., De Jongste, J. C., Sturkenboom, M. C. J. M. & Verhamme, K. M. C. Medication adherence and the risk of severe asthma exacerbations: a systematic review. *Eur Respir J* **45,** 396–407 (2015).
31. Papi, A. *et al.* Relationship of Inhaled Corticosteroid Adherence to Asthma Exacerbations in Patients with Moderate-to-Severe Asthma. *J. Allergy Clin. Immunol. Pract.* (2018). doi:10.1016/j.jaip.2018.03.008
32. McCarville, M., Sohn, M. W., Oh, E., Weiss, K. & Gupta, R. Environmental tobacco smoke and asthma exacerbations and severity: The difference between measured and reported exposure. *Arch. Dis. Child.* **98,** 510–514 (2013).
33. Marquette, C. H. *et al.* Long-term Prognosis of Near-Fatal Asthma. *Am. Rev. Respir. Dis.* **146,** 76–81 (1992).
34. Price, D. *et al.* Predicting frequent asthma exacerbations using blood eosinophil count and other patient data routinely available in clinical practice. *J. Asthma Allergy* 1 (2016). doi:10.2147/JAA.S97973
35. Black, M. H., Zhou, H., Takayanagi, M., Jacobsen, S. J. & Koebnick, C. Increased asthma risk and asthma-related health care complications associated with childhood obesity. *Am. J. Epidemiol.* **178,** 1120–1128 (2013).
36. Schatz, M. *et al.* Overweight/obesity and risk of seasonal asthma exacerbations. *J. Allergy Clin. Immunol. Pract.* **1,** 618–622 (2013).
37. Quinto, K. B. *et al.* The association of obesity and asthma severity and control in children. *J. Allergy Clin. Immunol.* **128,** 964–969 (2011).
38. Stingone, J. A., Ramirez, O. F., Svensson, K. & Claudio, L. Prevalence, demographics, and health outcomes of comorbid asthma and overweight in urban children. *J. Asthma* **48,** 876–885 (2011).
39. Sarpong, S. B. & Karrison, T. Sensitization to indoor allergens and the risk for asthma hospitalization in children. *Ann. Allergy, Asthma Immunol.* **79,** 455–459 (1997).
40. Stingone, J. A. & Claudio, L. Disparities in the use of urgent health care services among asthmatic children. *Ann. Allergy, Asthma Immunol.* **97,** 244–250 (2006).
41. Schatz, M., Cook, E. F., Joshua, A. & Petitti, D. Risk Factors for Asthma

For peer review only

Hospitalizations in a Managed Care Organization: Development of a Clinical Prediction Rule. *Am. J. Manag. Care* **9,** 538–547 (2003).
42. Rosas-Salazar, C. *et al.* Parental numeracy and asthma exacerbations in Puerto Rican children. *Chest* **144,** 92–98 (2013).
43. Bossios, A. & Papadopoulos, N. Viruses and asthma exacerbations. *Breathe* **3,** 51–58 (2006).
44. Leung, D. Y. M., Ledford, D. K., Jackson, D. J., Johnston, S. L. & London, P. The role of viruses in acute exacerbations of asthma. *J. Allergy Clin. Immunol.* **125,** 1178–1187 (2010).
45. Busse, W. W., Lemanske, R. F. & Gern, J. E. The Role of Viral Respiratory Infections in Asthma and Asthma Exacerbations NIH Public Access. *Lancet* **376,** 826–834 (2010).
46. King, G., Zeng, L. & King, G. Logistic Regression in Rare Events Data. *Polit. Anal.* **9,** 137–163 (2001).
47. Lieu, T. A., Quesenberry, C. P., Sorel, M. E., Mendoza, G. R. & Leong, A. B. Computer-based models to identify high-risk children with asthma. *Am J Respir Crit Care Med* (1998). doi:10.1164/ajrccm.157.4.9708124
48. Smith, J. R. *et al.* The at-risk registers in severe asthma (ARRISA) study: A cluster-randomised controlled trial examining effectiveness and costs in primary care. *Thorax* **67,** 1052–1060 (2012).
49. Van Vliet, D. *et al.* Prediction of asthma exacerbations in children by innovative exhaled inflammatory markers: Results of a longitudinal study. *PLoS One* **10,** 1–15 (2015).
50. Hallit, S. *et al.* Development of an asthma risk factors scale (ARFS) for risk assessment asthma screening in children. *Pediatr. Neonatol.* (2018). doi:10.1016/j.pedneo.2018.05.009
51. Forno, E. *et al.* Risk factors and predictive clinical scores for asthma exacerbations in childhood. *Chest* **138,** 1156–1165 (2010).
52. Finkelstein, J. & Jeong, I. cheol. Machine learning approaches to personalize early prediction of asthma exacerbations. *Ann. N. Y. Acad. Sci.* **1387,** 153–165 (2017).
53. Soyiri, I. N. *et al.* Improving predictive asthma algorithms with modelled environment data for Scotland: an observational cohort study protocol. *BMJ Open* **8,** e23289 (2018).
54. Simpson, C. R. *et al.* Evaluating the effectiveness, impact and safety of live attenuated and seasonal inactivated influenza vaccination: protocol for the Seasonal Influenza Vaccination Effectiveness II (SIVE II) study. *BMJ Open* **7,** e014200 (2017).
55. Simpson, C. R. *et al.* Seasonal Influenza Vaccination Effectiveness II (SIVE II): an observational study to evaluate live attenuated and trivalent inactivated influenza vaccination effectiveness, public health impact and safety – 2010/11 to 2015/16 seasons. *Heal. Technol Assess.* (in press)
56. Reddel, H. K. *et al.* An official American Thoracic Society/European Respiratory Society statement: Asthma control and exacerbations - Standardizing endpoints for clinical asthma trials and clinical practice. *Am. J. Respir. Crit. Care Med.* **180,** 59–99 (2009).
57. Scottish Government National Statistics Publications. *Introducing The Scottish Index of Multiple Deprivation 2016*. (2016).
58. Scottish Government. *Scottish Government Urban Rural Classification 2016.*
59. Scottish Government. *Review of Nomenclature of Units for Territorial Statistics (NUTS) Boundaries*. (2016).
60. British Thoracic Society. *British Guideline on the Management of Asthma: Quick Reference Guide*. *Scottish Intercollegiate Guidelines Network* (2016).

For peer review only

doi:10.1136/thx.2008.097741

61. Lewis, J. D. & Brensinger, C. Agreement between GPRD smoking data: A survey of general practitioners and a population-based survey. *Pharmacoepidemiol. Drug Saf.* **13,** 437–441 (2004).
62. Marston, L. *et al.* Issues in multiple imputation of missing data for large general practice clinical databases. *Pharmacoepidemiol. Drug Saf.* **19,** 618–626 (2010).
63. Deyo, R. A., Cherkin, D. C. & Ciol, M. A. Adapting a clinical comorbidity index for use with ICD-9-CM administrative databases. *J. Clin. Epidemiol.* **45,** 613–619 (1992).
64. Charlson, M. E., Pompei, P., Ales, K. L. & MacKenzie, R. A new method of classifying prognostic in longitudinal studies: development and validation. *Journal of Chronic Diseases* **40,** 373–383 (1987).
65. Blakey, J. D. *et al.* Identifying Risk of Future Asthma Attacks Using UK Medical Record Data: A Respiratory Effectiveness Group Initiative. *J. Allergy Clin. Immunol. Pract.* **5,** 1015–1024.e8 (2017).
66. Hess, L. M., Raebel, M. A., Conner, D. A. & Malone, D. C. Measurement of Adherence in Pharmacy Administrative Databases: A Proposal for Standard Definitions and Preferred Measures. *Ann. Pharmacother.* **40,** 1280–1288 (2006).
67. Wolpert, D. H. & Macready, W. G. No free lunch theorems for optimization. *IEEE Trans. Evol. Comput.* **1,** 67–82 (1997).
68. He, H. & Garcia, E. A. Learning from Imbalanced Data. *IEEE Trans. Knowl. DATA Eng.* **21,** 1263–1284 (2009).
69. Chawla, N. V., Bowyer, K. W., Hall, L. O. & Kegelmeyer, W. P. SMOTE: Synthetic Minority Over-sampling Technique. *J. Artif. Intell. Res.* **16,** 321–357 (2002).
70. Chicco, D. Ten quick tips for machine learning in computational biology. *BioData Min.* **10,** 1–17 (2017).
71. Kiefer, J. Sequential minimax search for a maximum. *Proc. Am. Math. Soc.* (1953). doi:10.2307/2032161
72. Hripcsak, G. & Rothschild, A. S. Agreement, the F-measure, and reliability in information retrieval. *J. Am. Med. Informatics Assoc.* **12,** 296–298 (2005).
73. Collins, G. S., Reitsma, J. B., Altman, D. G. & Moons, K. G. Transparent reporting of a multivariable prediction model for individual prognosis or diagnosis (TRIPOD): the TRIPOD Statement. *BMC Med.* (2015). doi:10.1186/s12916-014-0241-z
74. Nicholls, S. G. *et al.* The REporting of studies Conducted using Observational Routinely-collected health Data (RECORD) statement: Methods for arriving at consensus and developing reporting guidelines. *PLoS One* **10,** 1–23 (2015).

For peer review only

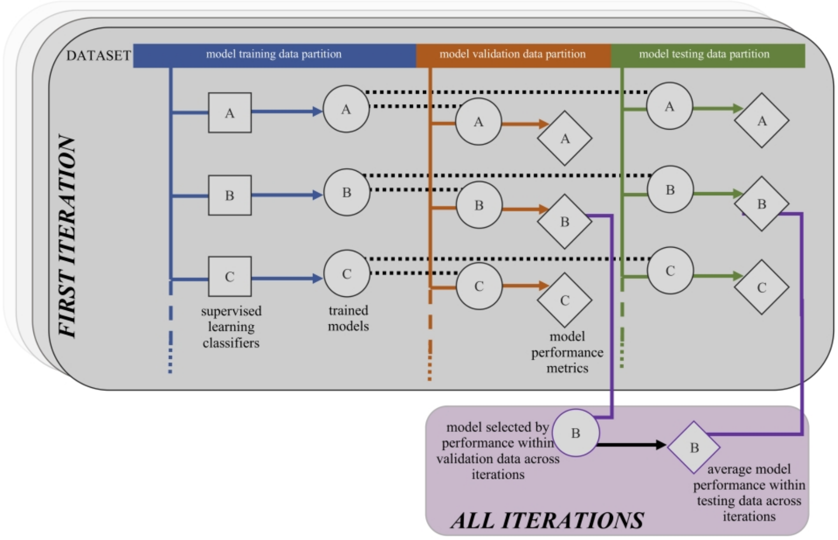

Process of selecting the highest performing model from the validation data, and the average performance of this model across iterations in the testing dataset. In the foreground we have the first iteration. We will use 100 iterations for statistical confidence, randomly permuting the data into training, validation, and testing subsets in each iteration

297x209mm (300 x 300 DPI)

Appendix A – Data harmonisation plan

Variable	Derivation dataset (LHS) format	External (unseen; SIVE II) dataset format	Harmonised format
Sex	Character – "M", "F" and "I" (less than 0.001% of records)	Character – "M", "F"	Character – "M", "F" and "I"
Birthday	Age (integer) at data extraction date (31st March 2018) or deduction date (indicated)	YYYY-MM-DD date format, all days set to 01 (true day redacted)	Age on March 31st, 2015 (approximate)
Scottish Index of Multiple Deprivation	Quintiles, 2012 and 2009 values	Deciles, 2012 values	Quintiles, 2012 values
Scottish Government Urban Rural Classification Scale	6-fold scale, from (1) Large Urban Areas to (6) Remote Rural Areas	8-fold scale, from (1) Large Urban Areas to (8) Very Remote Rural Areas	6-fold scale, from (1) Large Urban Areas to (6) Remote Rural Areas, 8-fold scale recoded as follows:<br>1 > 1<br>2 > 2<br>3 > 3<br>4, 5 > 4<br>6 > 5<br>7,8 > 6
Cause of death	ICD10 coded primary field, and 10 secondary cause fields	ICD10 coded primary field, and 10 secondary cause fields	*Aligned*
A&E cause of presentation	Presenting complaint free text field and 3 ICD10 coded disease fields	Presenting complaint free text field and 3 ICD10 coded disease fields	*Aligned*
Primary care records	Read Codes (version 2)	Read Codes (version 2)	*Aligned*
Primary care prescriptions	Standardised [a] text drug name and dose fields	Standardised [a] text drug name and dose fields	*Aligned*
Hospital inpatient admission records	N/A	ICD10 coded primary field, and 5 secondary cause fields	*Omitted as alignment not possible*
Event Date	Standardised date format	Standardised date format	*Aligned*

[a] Auto-fill assisted free text field

For peer review only

Appendix B – Machine Learning classifier hyperparameters

Naïve Bayes Classifier
Implemented using the r function *naivebayes*, from the package of the same name [72].
No hyperparameters.

Support Vector Machine
Implemented using the r function *svm*, from the package *e1071* [73] *which builds upon the LIBSVM package* [74], using a radial basis kernel function.

- *GAMMA* = Radial basis kernel function gamma parameter, corresponding to the kernel bandwidth (default 1/k): $2^{(-5:10)}$
- *COST* = Cost of constraints violation, i.e. samples penalised when crossing the boundary (default 1): $2^{(-5:10)}$

Ensemble: Bagging
Bagging methods learn from multiple models which are staged in parallel.
Random Forests
Implemented using the r function *randomForest*, from the package of the same name [75].

- *NTREE* = Number of trees to grow (default 500): 500, 750, 1000
- *MTRY* = Number of variables randomly sampled as candidates at each split (default square root of the number of predictors; k): floor($0.5 * \sqrt{k}$), floor($\sqrt{k}$), floor($2*\sqrt{k}$) – in which floor represents the rounded-down integer value.

Ensemble: Boosting
Learning from multiple models which are staged *sequentially*, usually tree-based, constructed from different subsamples of the training dataset.
Extreme Gradient Boosting
Implemented using the r package *xgboost* [76], with 10-fold cross validation, repeated 3 times.

- NROUNDS = maximum number of iterations (default 100): 50,100
- MAXDEPTH = Maximum depth of each tree (default = 6): (1:5)^2
- ETA = step size of each boosting step (default = 0.3): 0.25, 0.5, 1

Ensemble: Stacking
Combining models from different classifiers, with an over-arching supervisor model which determines the best way to use all sources of information for prediction. The base set of weak learners will comprise all aforementioned model and hyperparameter combinations, and the meta-learner (random forest with 500 trees and mtry = floor($0.5 * \sqrt{k}$)) will use all weak learners with a validation set performance in the top 50%.

For peer review only

TRIPOD Checklist: Prediction Model Development and Validation

Section/Topic	Item		Checklist Item	Page
**Title and abstract**				
Title	1	D;V	Identify the study as developing and/or validating a multivariable prediction model, the target population, and the outcome to be predicted.	1* (* main paper will include validation)
Abstract	2	D;V	Provide a summary of objectives, study design, setting, participants, sample size, predictors, outcome, statistical analysis, results, and conclusions.	2** (** more thorough in main paper)
**Introduction**				
Background and objectives	3a	D;V	Explain the medical context (including whether diagnostic or prognostic) and rationale for developing or validating the multivariable prediction model, including references to existing models.	4
	3b	D;V	Specify the objectives, including whether the study describes the development or validation of the model or both.	5
**Methods**				
Source of data	4a	D;V	Describe the study design or source of data (e.g., randomized trial, cohort, or registry data), separately for the development and validation data sets, if applicable.	5
	4b	D;V	Specify the key study dates, including start of accrual; end of accrual; and, if applicable, end of follow-up.	6
Participants	5a	D;V	Specify key elements of the study setting (e.g., primary care, secondary care, general population) including number and location of centres.	5
	5b	D;V	Describe eligibility criteria for participants.	5-6
	5c	D;V	Give details of treatments received, if relevant.	-
Outcome	6a	D;V	Clearly define the outcome that is predicted by the prediction model, including how and when assessed.	6
	6b	D;V	Report any actions to blind assessment of the outcome to be predicted.	-
Predictors	7a	D;V	Clearly define all predictors used in developing or validating the multivariable prediction model, including how and when they were measured.	7-8**
	7b	D;V	Report any actions to blind assessment of predictors for the outcome and other predictors.	-
Sample size	8	D;V	Explain how the study size was arrived at.	6
Missing data	9	D;V	Describe how missing data were handled (e.g., complete-case analysis, single imputation, multiple imputation) with details of any imputation method.	7-8**
Statistical analysis methods	10a	D	Describe how predictors were handled in the analyses.	8-9
	10b	D	Specify type of model, all model-building procedures (including any predictor selection), and method for internal validation.	8-9
	10c	V	For validation, describe how the predictions were calculated.	8-9
	10d	D;V	Specify all measures used to assess model performance and, if relevant, to compare multiple models.	8-9
	10e	V	Describe any model updating (e.g., recalibration) arising from the validation, if done.	-**
Risk groups	11	D;V	Provide details on how risk groups were created, if done.	-
Development vs. validation	12	V	For validation, identify any differences from the development data in setting, eligibility criteria, outcome, and predictors.	9**
**Results**				
Participants	13a	D;V	Describe the flow of participants through the study, including the number of participants with and without the outcome and, if applicable, a summary of the follow-up time. A diagram may be helpful.	Protocol Paper
	13b	D;V	Describe the characteristics of the participants (basic demographics, clinical features, available predictors), including the number of participants with missing data for predictors and outcome.	Protocol Paper
	13c	V	For validation, show a comparison with the development data of the distribution of important variables (demographics, predictors and outcome).	Protocol Paper
Model development	14a	D	Specify the number of participants and outcome events in each analysis.	Protocol Paper
	14b	D	If done, report the unadjusted association between each candidate predictor and outcome.	Protocol Paper
Model specification	15a	D	Present the full prediction model to allow predictions for individuals (i.e., all regression coefficients, and model intercept or baseline survival at a given time point).	Protocol Paper
	15b	D	Explain how to the use the prediction model.	Protocol Paper
Model performance	16	D;V	Report performance measures (with CIs) for the prediction model.	Protocol Paper
Model-updating	17	V	If done, report the results from any model updating (i.e., model specification, model performance).	Protocol Paper
**Discussion**				
Limitations	18	D;V	Discuss any limitations of the study (such as nonrepresentative sample, few events per predictor, missing data).	Protocol Paper
Interpretation	19a	V	For validation, discuss the results with reference to performance in the development data, and any other validation data.	Protocol Paper
	19b	D;V	Give an overall interpretation of the results, considering objectives, limitations, results from similar studies, and other relevant evidence.	Protocol Paper
Implications	20	D;V	Discuss the potential clinical use of the model and implications for future research.	Protocol Paper
**Other information**				
Supplementary information	21	D;V	Provide information about the availability of supplementary resources, such as study protocol, Web calculator, and data sets.	Protocol Paper
Funding	22	D;V	Give the source of funding and the role of the funders for the present study.	Abstract

For peer review only

TRIPOD Checklist: Prediction Model Development and Validation

*Items relevant only to the development of a prediction model are denoted by D, items relating solely to a validation of a prediction model are denoted by V, and items relating to both are denoted D;V. We recommend using the TRIPOD Checklist in conjunction with the TRIPOD Explanation and Elaboration document.

For peer review only
